# DanGer shock criteria and outcomes in acute myocardial infarction-related cardiogenic shock treated with Impella: the J-PVAD registry

**DOI:** 10.1093/eurheartj/ehaf787

**Published:** 2025-10-13

**Authors:** Riku Arai, Keisuke Kojima, Daisuke Fukamachi, Yasuo Okumura

**Affiliations:** Division of Cardiology, Department of Medicine, Nihon University School of Medicine, Oyaguchikamicho 30-1, Tokyo 1738610, Japan; Division of Cardiology, Department of Medicine, Nihon University School of Medicine, Oyaguchikamicho 30-1, Tokyo 1738610, Japan; Division of Cardiology, Department of Medicine, Nihon University School of Medicine, Oyaguchikamicho 30-1, Tokyo 1738610, Japan; Division of Cardiology, Department of Medicine, Nihon University School of Medicine, Oyaguchikamicho 30-1, Tokyo 1738610, Japan

**Keywords:** Cardiogenic shock, Eligibility, Impella, Mortality, VA-ECMO

## Abstract

**Background and Aims:**

The DanGer shock trial showed a survival benefit of Impella in highly selected patients with ST-elevation myocardial infarction-related cardiogenic shock (STEMI-CS). In real-world settings, however, Impella is often used in broader populations not meeting trial criteria. This study aimed to assess the distribution and outcomes of DanGer shock-eligible and -ineligible acute myocardial infarction-related cardiogenic shock (AMI-CS) patients in a nationwide Japanese registry.

**Methods:**

A total of 3975 AMI-CS patients treated with Impella between 2020 and 2023 were identified from the J-PVAD registry. Patients were stratified into five groups: eligible STEMI-CS, non-eligible STEMI-CS, out-of-hospital cardiac arrest (OHCA), mechanical complications (MCs), and non-STEMI-CS (NSTEMI-CS). Eligible STEMI-CS was defined as STEMI-CS fulfilling all of the following: lactate ≥ 2.5 mmol/L, systolic blood pressure <100 mmHg or catecholamine use, left ventricular ejection fraction <45%, and shock onset to first Impella support ≤24 h. Thirty-day mortality, associated risk factors, and complications were examined across the five groups.

**Results:**

Eligible STEMI-CS accounted for 35.6% of all AMI-CS cases, with a 30-day mortality of 37.6%. Non-eligible STEMI-CS patients had lower mortality (27.6%), but it increased significantly with advanced age, renal dysfunction, veno-arterial extracorporeal membrane oxygenation use, ventricular arrhythmia, or sepsis. Thirty-day mortality was 51.3% in OHCA (63.4% without return of spontaneous circulation), 39.8% in MC, and 33.3% in NSTEMI-CS groups.

**Conclusions:**

In this nationwide registry, one-third of AMI-CS patients treated with Impella met DanGer shock criteria. Outcomes in non-eligible subgroups were heterogeneous, highlighting the importance of individualized risk assessment in guiding Impella use in real-world practice.

## Structured Graphical Abstract

**Structured Graphical Abstract:**
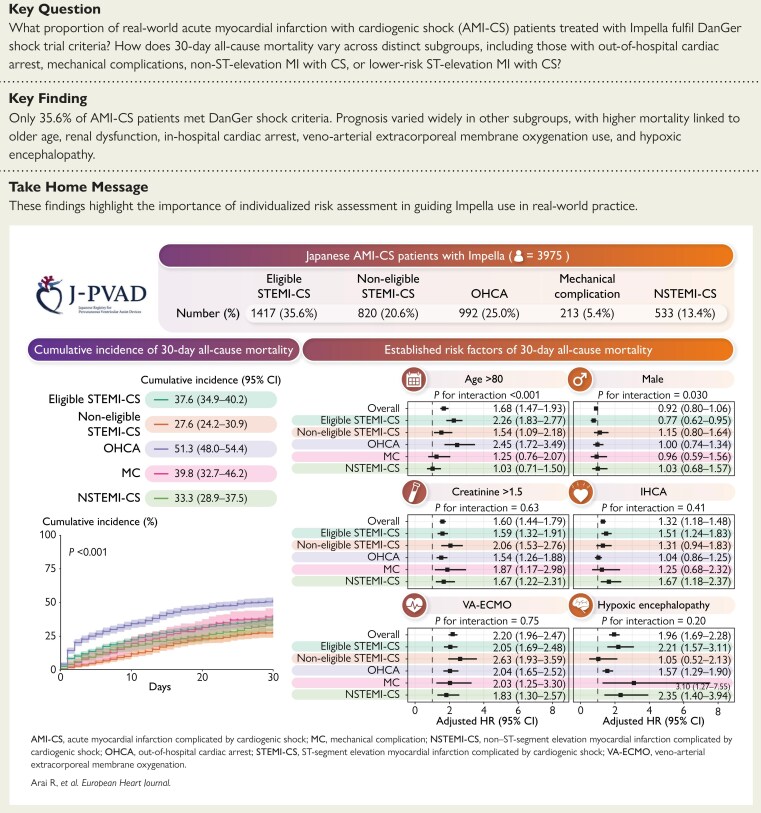

**See the editorial comment for this article ‘DanGerous real-world practice of percutaneous ventricular assist devices’, by H. Thiele and A. Freund, https://doi.org/10.1093/eurheartj/ehaf966.**

## Introduction

Cardiogenic shock (CS) complicates approximately 5%–10% of acute myocardial infarction (AMI) cases and is associated with high early mortality, with rates ranging from 30% to 50%.^1,2^ While mechanical circulatory support (MCS) devices are widely used in AMI complicated by CS (AMI-CS), neither intra-aortic balloon pump (IABP) nor veno-arterial extracorporeal membrane oxygenation (VA-ECMO) has demonstrated a clear mortality benefit.^3,4^

The recent DanGer shock trial demonstrated improved 180-day mortality with Impella compared to conventional care in carefully selected patients with ST-elevation myocardial infarction (STEMI).^5^ However, the applicability of these findings to real-world practice remains uncertain. Data suggest that only 22.4% of AMI-CS patients meet DanGer shock eligibility, while in practice, Impella is often used in broader populations—including those with the Society for Cardiovascular Angiography and Interventions (SCAI) shock stage B shock, out-of-hospital cardiac arrest (OHCA), mechanical complications (MC), or non-STEMI (NSTEMI)-CS.^6,7^ The outcomes of such patients remain insufficiently characterized.

In addition to eligibility criteria, prior studies have identified advanced age (>80 years), in-hospital cardiac arrest (IHCA), renal dysfunction, and the use of VA-ECMO as strong predictors of mortality.^8–11^ Moreover, complications related to Impella itself may further impact outcomes.^12^ Thus, a comprehensive understanding of both baseline risks and procedural complications is critical when considering Impella therapy.

To better define real-world outcomes in this context, data from the Japanese Percutaneous Ventricular Assist Device (J-PVAD) registry—a nationwide, prospective, multicentre registry in Japan that includes all patients treated with Impella for drug-refractory heart failure—were analysed. This study aimed to assess mortality in AMI-CS patients according to DanGer shock eligibility, established risk factors, and complications, to better characterize the real-world role of Impella in this high-risk population.

## Methods

### Study population

This was a *post hoc* analysis of the J-PVAD registry, a nationwide multicentre database of patients with drug-refractory heart failure treated with Impella in Japan. Patients who received Impella for AMI-CS between February 2020 and December 2023 were analysed. The design and patient enrolment of the J-PVAD registry have been described previously.^13^ Patients were excluded if Impella implantation was unsuccessful or if follow-up data were missing.

The J-PVAD registry was approved by the ethics committee of each participating institution. Written informed consent was waived because the data were obtained during routine clinical care and were fully anonymized, in accordance with ethical guidelines and the Declaration of Helsinki.

### Pragmatic DanGer shock criteria

In the DanGer shock trial, patients with OHCA accompanied by coma and those with MCs were excluded, while NSTEMI-CS patients were not included. Accordingly, patients whose initial presentation was OHCA were first classified into the OHCA group, regardless of neurological status upon hospital arrival. Next, patients were classified into the MC group if the primary indication for Impella support was a MC, defined as cardiac rupture, ventricular septal rupture, or papillary muscle rupture. Subsequently, patients whose initial presentation was not STEMI were classified into the NSTEMI-CS group. All other patients were categorized into the STEMI-CS group.

The Pragmatic DanGer shock criteria were defined as the fulfilment of all four of the following conditions, regardless of the underlying AMI-CS subtype: (i) lactate level ≥ 2.5 mmol/L, (ii) systolic blood pressure < 100 mmHg or the use of catecholamines, (iii) left ventricular ejection fraction (LVEF) < 45%, and (iv) time from shock onset to first Impella support ≤ 24 h.

Among patients classified into the STEMI-CS group, those who met all four of these criteria were assigned to the Eligible STEMI-CS group, representing a population equivalent to that included in the DanGer shock trial. STEMI-CS patients who did not fulfil all four criteria were categorized as non-eligible STEMI-CS.

Details regarding how the DanGer shock exclusion criteria were operationalized in the J-PVAD registry are provided in online supplementary data online, *Table S1*.

### Definitions and endpoints

CS was defined as (i) prolonged hypotension (systolic blood pressure ≤ 90 mmHg) lasting at least 30 min or a cardiac index ≤ 2.2 L/min/m² despite adequate fluid replacement, in order to differentiate it from hypovolaemia and septic shock, or (ii) a condition requiring the use of intravenous vasoactive inotropes and/or MCS to maintain a systolic blood pressure ≥90 mmHg.

Thirty-day mortality was defined as death from any cause within 30 days after Impella implantation, with vital status at 30 days confirmed through in-hospital follow-up, chart review, or contact via telephone or letter. Cardiac death was defined as any death directly attributable to cardiac causes, including AMI, low-output syndrome, and ventricular arrhythmia, as well as unwitnessed deaths, deaths of undetermined cause, and any death related to Impella insertion or other procedural complications. Non-cardiac death was defined as any death not meeting the criteria for cardiac death, and included both vascular causes and non-cardiovascular causes. Major bleeding was defined as a haemorrhage requiring surgical intervention or transfusion, or intracranial bleeding, and/or a haematoma larger than 5 cm in diameter or requiring surgical intervention. Of these, bleeding events considered by the attending physician to be associated with Impella insertion were defined as Impella-related bleeding. Limb ischaemia was defined as newly developed lower limb hypoperfusion requiring treatment, characterized by symptoms such as decreased skin temperature of the lower extremities or diminished peripheral pulses. Worsening renal failure was defined according to the Kidney Disease: Improving Global Outcomes (KDIGO) guideline^14^ as stage 3, including a three-fold increase in baseline serum creatinine, a serum creatinine level ≥4.0 mg/dL, initiation of renal replacement therapy, or urine output <0.3 mL/kg/h for 24 h. Ischaemic stroke was defined as a newly identified transient or persistent focal or global neurological deficit confirmed by standard neurological examination (including appropriate diagnostic tests and documentation of consultation with a neurologist or equivalent physician), along with radiological evidence of cerebral infarction. Ventricular arrhythmias were defined as ventricular fibrillation or sustained ventricular tachycardia, with the latter defined as a heart rate > 120 bpm, QRS duration > 110 ms, and duration >60 s. Sepsis was defined as a clinically evident infection treated with therapeutic (non-prophylactic) antimicrobial agents, accompanied by features such as pain, fever, drainage, leucocytosis, positive blood cultures, and systemic signs such as hypotension.

### Statistical analysis

Continuous variables were presented as medians with interquartile ranges (IQRs), and categorical variables were expressed as counts and percentages. Differences among the five patient groups (Eligible STEMI-CS, Non-eligible STEMI-CS, OHCA, MC, and NSTEMI-CS) were assessed using the Kruskal–Wallis test for continuous variables and the chi-square test for categorical variables, as appropriate.

Clinical events for the overall AMI-CS population and each of the five subgroups were reported as cumulative incidence at 30 days with 95% confidence intervals (CIs). Differences among the five groups were assessed using the log-rank test. The median follow-up period for each group was presented as the median with IQRs. Kaplan–Meier curves with 95% CIs were generated to evaluate 30-day mortality across the five groups, and differences were assessed using the log-rank test.

To evaluate time-to-event outcomes and adjust for potential confounders, multivariable Cox proportional hazards models were constructed for 30-day mortality in both the overall AMI-CS cohort and within each of the five subgroups. Two models were developed: Model 1 included fundamental patient characteristics, established prognostic factors, and treatment interventions, and was adjusted for age > 80 years, creatinine > 1.5 mg/dL, IHCA, use of VA-ECMO, male sex, hypoxic encephalopathy, percutaneous coronary intervention (PCI), coronary artery bypass grafting (CABG), non-CABG surgery, and the presence of pragmatic DanGer shock criteria. Model 2 focused on the association between major complications and 30-day mortality and was adjusted for age > 80 years, male sex, and the occurrence of major complications (major bleeding, limb ischaemia, worsening renal failure, ischaemic stroke, ventricular arrhythmia, and sepsis).

Adjusted hazard ratios (HRs) with 95% CIs from each multivariable model were visualized in forest plots for both the overall AMI-CS population and each subgroup. Variables related to major complications were derived from Model 2, while all other variables were based on Model 1. To evaluate heterogeneity across the five subgroups, *P* for interaction values were calculated for each variable.

Model performance was evaluated using the concordance index (*c*-statistics). Multicollinearity among covariates was assessed by calculating the variance inflation factor (VIF) and condition index across the Cox regression models.

Subgroup analyses explored differences in baseline characteristics and clinical events between: (i) Impella-first vs VA-ECMO-first patients (defined as those receiving VA-ECMO prior to Impella) in the Eligible STEMI-CS group; (ii) patients with vs without pre-hospital return of spontaneous circulation (ROSC) in the OHCA group; and (iii) patients aged ≤80 vs > 80 years in the MC group.

Differences between these paired subgroups were tested using the Mann–Whitney U test for continuous variables and the chi-square test for categorical variables.

If one or more of the four pragmatic DanGer shock criteria were missing, the respective criterion or criteria were conservatively considered fulfilled (i.e. eligible).

All statistical analyses were performed using R version 4.4.1 (The R Foundation for Statistical Computing, Vienna, Austria). A two-sided *P* value < .05 was considered statistically significant.

## Results

### Patient selection and subgroup classification

**Figure 1:**
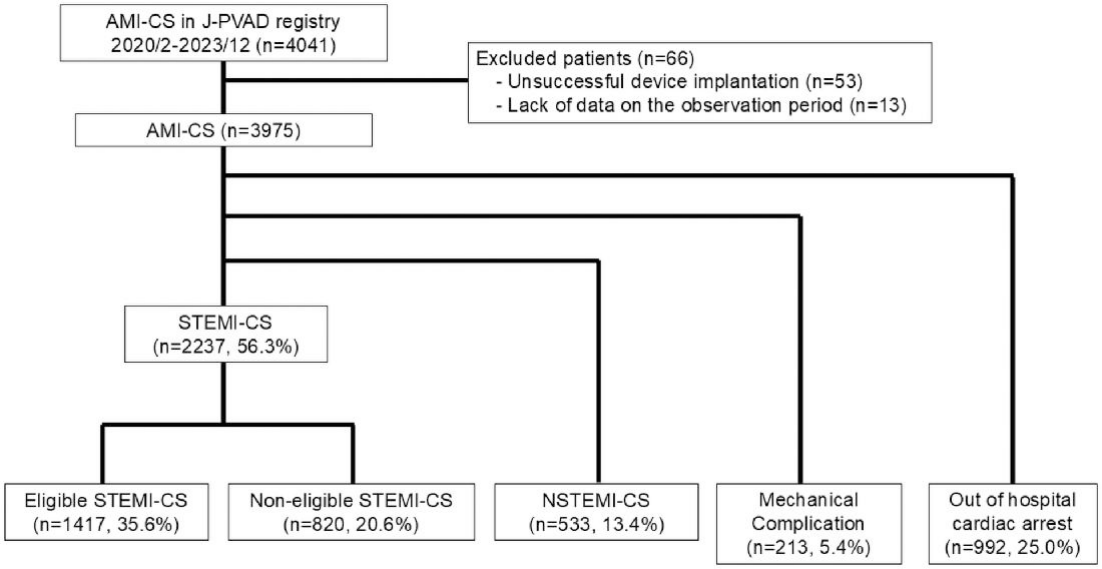
Study flow and patient classification. Among 4041 patients with AMI-CS enrolled in the J-PVAD registry between February 2020 and December 2023, a total of 66 patients (1.6%) were excluded due to unsuccessful Impella implantation (*n* = 53) or missing follow-up data (*n* = 13). The remaining 3975 patients were classified into five groups: STEMI-CS (*n* = 2,237, 56.3%), NSTEMI-CS (*n* = 533, 13.4%), MC (*n* = 213, 5.4%), and OHCA (*n* = 992, 25.0%). The STEMI-CS group was further subdivided into Eligible STEMI-CS (*n* = 1,417, 35.6%) and Non-eligible STEMI-CS (*n* = 820, 20.6%) based on the fulfilment of the pragmatic DanGer shock criteria. Abbreviations: AMI-CS, acute myocardial infarction complicated by cardiogenic shock; J-PVAD, Japanese Percutaneous Ventricular Assist Device; NSTEMI-CS, non-ST-elevation myocardial infarction complicated by cardiogenic shock; OHCA, out-of-hospital cardiac arrest; STEMI-CS, ST-elevation myocardial infarction complicated by cardiogenic shock

Of 4041 patients in the J-PVAD registry, 66 were excluded (53 for unsuccessful implantation, 13 for missing follow-up data), leaving 3975 AMI-CS patients for analysis. They were classified into Eligible STEMI-CS (*n* = 1417, 35.6%), Non-eligible STEMI-CS (*n* = 820, 20.6%), OHCA (*n* = 992, 25.0%), MC (*n* = 213, 5.4%), and NSTEMI-CS (*n* = 533, 13.4%) (*Figure 1*).

### Baseline characteristics

Baseline and procedural data are shown in *Table 1*. Median age was 71.0 years, 16.7% were over 80, and 80.7% were male. OHCA patients were the youngest (median 64.0), while MC and NSTEMI-CS were the oldest (median 75.0). Advanced age, heart failure, and chronic kidney disease (CKD) were more frequent in MC and NSTEMI-CS groups. The OHCA group exhibited the highest lactate levels, the lowest LVEF, and the highest prevalence of hypoxic encephalopathy. Catecholamine use was most common in Eligible STEMI-CS and OHCA, and VA-ECMO was used most in OHCA (62.7%). PCI was performed in 89.6% of patients, while both CABG and non-CABG surgery were most frequently performed in the MC group. Impella CP was used in 94.8% of patients. The door to first Impella support time and shock onset to first Impella support time were shortest in the Eligible STEMI-CS group and longest in the MC group. The pragmatic DanGer shock criteria were met by 81.3% of the OHCA group, 41.3% of the MC group, and 55.3% of the NSTEMI-CS group.

**Table 1:** Baseline characteristics, and procedure details

	All *N* = 3975	Eligible STEMI-CS *N* = 1417 (35.6%)	Non-eligible STEMI-CS *N* = 820 (20.6%)	OHCA *N* = 992 (25.0%)	MC *N* = 213 (5.4%)	NSTEMI-CS *N* = 533 (13.4%)	*P* value
Age, median (IQR)	71.0 (61.0–78.0)	72.0 (64.0–79.0)	71.0 (61.0–78.0)	64.0 (55.0–73.0)	75.0 (69.0–81.0)	75.0 (68.0–81.0)	<.001
Age > 80, *n* (%)	664/3975 (16.7)	265 (18.7)	141 (17.2)	56 (5.6)	54 (25.4)	148 (27.8)	<.001
Male, *n* (%)	3206/3975 (80.7)	1133 (80.0)	637 (77.7)	882 (88.9)	127 (59.6)	427 (80.1)	<.001
Body mass index, kg/m^2^, median (IQR)	23.5 (21.1–26.1)	23.3 (20.8–25.8)	23.4 (20.7–26.0)	24.2 (21.6–26.7)	23.1 (21.1–26.0)	23.5 (20.7–26.0)	<.001
Hypertension, *n* (%)	2472/3974 (62.2)	902 (63.7)	507 (61.8)	525 (52.9)	138 (64.8)	400 (75.2)	<.001
Dyslipidemia, *n* (%)	1836/3974 (46.2)	702 (49.5)	390 (47.6)	367 (37.0)	89 (41.8)	288 (54.1)	<.001
Diabetes mellitus, *n* (%)	1651/3974 (41.5)	610 (43.0)	321 (39.1)	338 (34.1)	83 (39.0)	299 (56.2)	<.001
Current smoking, *n* (%)	1250/3974 (31.5)	446 (31.5)	261 (31.8)	343 (34.6)	56 (26.3)	144 (27.1)	.016
Prior myocardial infarction, *n* (%)	734/3974 (18.5)	237 (16.7)	146 (17.8)	153 (15.4)	65 (30.5)	133 (25.0)	<.001
History of heart failure, *n* (%)	575/3974 (14.5)	168 (11.9)	121 (14.8)	99 (10.0)	45 (21.1)	142 (26.7)	<.001
Chronic kidney disease, *n* (%)	1102/3974 (27.7)	397 (28.0)	214 (26.1)	205 (20.7)	73 (34.3)	213 (40.0)	<.001
Haemodialysis, *n* (%)	146/3974 (3.7)	38 (2.7)	25 (3.0)	31 (3.1)	3 (1.4)	49 (9.2)	<.001
Prior stroke/TIA, *n* (%)	307/3974 (7.7)	107 (7.6)	59 (7.2)	64 (6.5)	17 (8.0)	60 (11.3)	.016
IHCA, *n* (%)	1375/3975 (34.6)	510 (36.0)	180 (22.0)	490 (49.4)	33 (15.5)	162 (30.4)	<.001
Systolic blood pressure, mmHg, median (IQR)	86.0 (69.0–107.0)	84.0 (68.0–98.0)	102.0 (80.3–122.0)	80.0 (50.0–102.0)	90.0 (72.0–104.0)	86.0 (70.0–107.8)	<.001
Systolic blood pressure < 100, mmHg, *n* (%)	2695/3975 (67.8)	1095 (77.3)	380 (46.3)	715 (72.1)	142 (66.7)	363 (68.1)	<.001
Diastolic blood pressure, mmHg, median (IQR)	58.0 (42.0–72.0)	55.0 (41.0–68.0)	65.0 (51.0–80.0)	53.0 (27.0–72.0)	58.0 (45.0–69.0)	56.0 (43.0–70.0)	<.001
Heart rate, bpm, median (IQR)	90.0 (70.0–110.0)	90.0 (68.0–110.0)	93.0 (75.0–110.0)	85.0 (60.0–104.0)	102.0 (83.5–116.0)	94.0 (75.0–110.0)	<.001
Creatinine, mg/dL, median (IQR)	1.2 (1.0–1.6)	1.2 (1.0–1.6)	1.1 (0.9–1.6)	1.2 (1.0–1.5)	1.4 (1.0–2.2)	1.3 (1.0–2.0)	<.001
Creatinine > 1.5 mg/dL, *n* (%)	1142/3907 (29.2)	392 (28.1)	218 (26.8)	233 (24.1)	94 (44.8)	205 (39.4)	<.001
Albumin, g/dL, median (IQR)	3.5 (3.0–3.9)	3.6 (3.2–4.0)	3.6 (3.1–4.1)	3.4 (2.8–3.8)	3.0 (2.7–3.4)	3.4 (2.9–3.8)	<.001
Lactate, mmol/L, median (IQR)	5.7 (2.9–10.5)	6.2 (4.2–9.7)	2.3 (1.7–4.3)	11.1 (6.9–15.0)	3.7 (2.0–6.6)	4.1 (2.0–7.3)	<.001
Lactate ≥ 2.5 mmol/L, *n* (%)	3354/3975 (84.4)	1417 (100)	438 (53.4)	941 (94.9)	159 (74.6)	399 (74.9)	<.001
LVEF, %, median (IQR)	30.0 (20.0–40.0)	25.0 (20.0–30.0)	32.0 (24.0–45.0)	20.0 (12.8–30.0)	45.0 (34.5–57.0)	30.0 (20.0–35.0)	<.001
LVEF < 45%, *n* (%)	3719/3975 (93.6)	1417 (100)	681 (83.0)	973 (98.1)	148 (69.5)	500 (93.8)	<.001
Catecholamine use at initiation of Impella support							
Epinephrine, *n* (%)	727/3975 (18.3)	225 (15.9)	68 (8.3)	343 (34.6)	27 (12.7)	64 (12.0)	<0.001
Dobutamine, *n* (%)	1107/3975 (27.8)	393 (27.7)	240 (29.3)	199 (20.1)	92 (43.2)	183 (34.3)	<0.001
Norepinephrine, *n* (%)	1973/3975 (49.6)	821 (57.9)	330 (40.2)	429 (43.2)	116 (54.5)	277 (52.0)	<.001
Dopamine, *n* (%)	392/3975 (9.9)	137 (9.7)	61 (7.4)	95 (9.6)	49 (23.0)	50 (9.4)	<.001
Any catecholamine, *n* (%)	3014/3975 (75.8)	1170 (82.6)	455 (55.5)	817 (82.4)	174 (81.7)	398 (74.7)	<.001
Use of pulmonary artery catheter, *n* (%)	2561/3975 (64.4)	905 (63.9)	521 (63.5)	646 (65.1)	140 (65.7)	349 (65.5)	.85
Hypoxic encephalopathy, *n* (%)	372/3975(9.4)	61 (4.3)	19 (2.3)	258 (26.0)	7 (3.3)	27 (5.1)	<.001
PCI, *n* (%)	3560/3975 (89.6)	1334 (94.1)	740 (90.2)	922 (92.9)	96 (45.1)	468 (87.8)	<.001
CABG, *n* (%)	296/3975 (7.4)	69 (4.9)	61 (7.4)	33 (3.3)	52 (24.4)	81 (15.2)	<.001
Non-CABG surgery, *n* (%)	392/3975 (9.9)	87 (6.1)	71 (8.7)	38 (3.8)	158 (74.2)	38 (7.1)	<.001
IABP, *n* (%)	570/3975 (14.3)	183 (12.9)	155 (18.9)	85 (8.6)	57 (26.8)	90 (16.9)	<.001
IABP first (use of IABP prior to Impella), *n* (%)	337/570 (59.1)	90 (49.2)	106 (68.4)	62 (72.9)	28 (49.1)	51 (56.7)	.024
VA-ECMO use, *n* (%)	1747/3975 (43.9)	594 (41.9)	231 (28.2)	622 (62.7)	108 (50.7)	192 (36.0)	<.001
VA-ECMO first (use of VA-ECMO prior to Impella), *n* (%)	1231/1747 (70.5)	355 (59.8)	145 (62.8)	557 (89.5)	50 (46.3)	124 (64.6)	<.001
Ventricular assist device, *n* (%)	12/3975 (0.3)	4 (0.3)	4 (0.5)	3 (0.3)	1 (0.5)	0 (0)	.44
Devise type of first Impella							
Impella 2.5, *n* (%)	96/3975 (2.4)	27 (1.9)	25 (3.0)	24 (2.4)	6 (2.8)	14 (2.6)	<.001
Impella CP, *n* (%)	3770/3975 (94.8)	1379 (97.3)	755 (92.1)	950 (95.8)	185 (86.9)	501 (94.0)	
Impella 5.0, *n* (%)	51/3975 (1.3)	6 (0.4)	23 (2.8)	8 (0.8)	8 (3.8)	6 (1.1)	
Impella 5.5, *n* (%)	58/3975 (1.5)	5 (0.4)	17 (2.1)	10 (1.0)	14 (6.6)	12 (2.3)	
Door to first Impella support, hour, median (IQR)	2.2 (1.3–4.7)	1.8 (1.2–3.2)	2.2 (1.3–10.6)	2.0 (1.2–3.2)	4.7 (2.1–23.5)	4.0 (2.1–18.2)	<.001
Shock onset to first Impella support, hour, median (IQR)	5.3 (2.8–10.3)	4.0 (2.1–6.8)	6.0 (2.3–33.8)	6.3 (4.2–9.8)	10.3 (5.0–31.1)	6.0 (2.7–13.4)	<.001
Shock onset to first Impella support ≤24 h, *n* (%)	3531/3975 (88.8)	1417 (100)	590 (72.0)	906 (91.3)	160 (75.1)	458 (85.9)	<.001
Impella assist time, hour, median (IQR)	93.2 (43.7–165.0)	96.1 (45.5–165.3)	83.8 (44.3–160.0)	91.7 (42.2–159.2)	111.6 (29.4–192.1)	93.4 (44.1–162.3)	.22
Pragmatic DanGer shock criteria	2606/3975 (65.6)	1417 (100)	0 (0)	806 (81.3)	88 (41.3)	295 (55.3)	<.001

Abbreviations: CABG, coronary artery bypass grafting; IABP, intra-aortic balloon pump; IHCA, in-hospital cardiac arrest; IQR, interquartile range; LVEF, left ventricular ejection fraction; MC, mechanical complication; NSTEMI-CS, non-ST-segment elevation myocardial infarction complicated by cardiogenic shock; OHCA, out-of-hospital cardiac arrest; PCI, percutaneous coronary intervention; STEMI-CS, ST-segment elevation myocardial infarction complicated by cardiogenic shock; TIA, transient ischaemic attack; VA-ECMO, veno-arterial extracorporeal membrane oxygenation.

Data are presented as *n* (%), or median (interquartile range) unless otherwise indicated. *P* values were calculated using the Kruskal–Wallis test for continuous variables and the chi-square test for categorical variables to compare differences across the five groups.

### Clinical events in the overall AMI-CS population and subgroups

**Figure 2:**
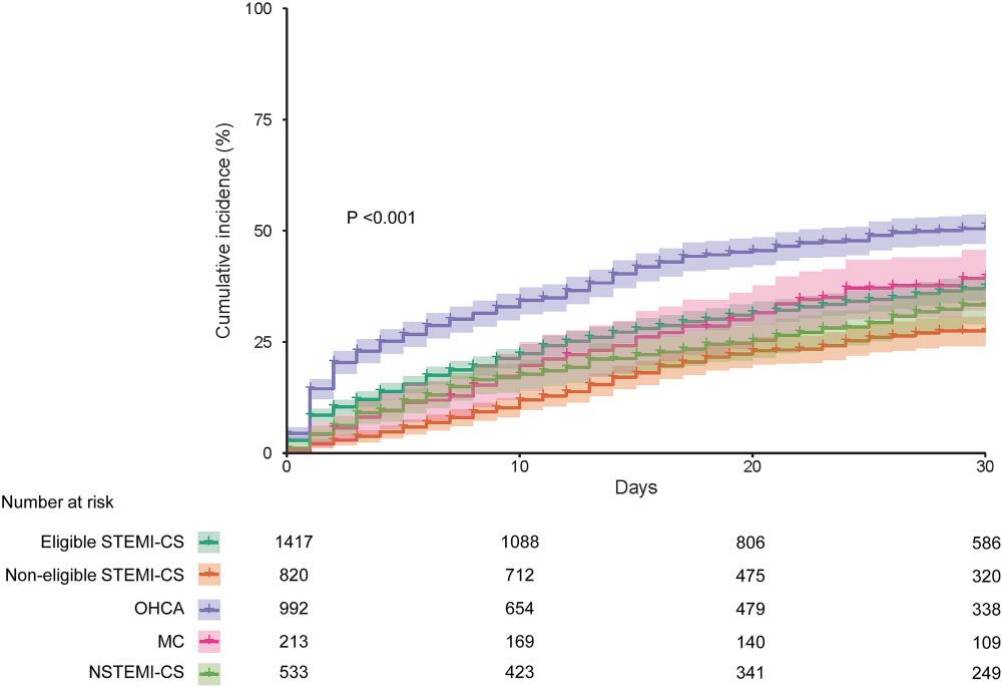
Kaplan–Meier curves for 30-day all-cause mortality in five AMI-CS subgroups. Kaplan–Meier estimates of cumulative incidence of death from any cause at 30 days were 37.6% (95% CI: 34.9–40.2) in the Eligible STEMI-CS group, 27.6% (95% CI: 24.2–30.9) in the Non-eligible STEMI-CS group, 51.3% (95% CI: 48.0–54.4) in the OHCA group, 39.8% (95% CI: 32.7–46.2) in the MC group, and 33.3% (95% CI: 28.9–37.5) in the NSTEMI-CS group (log-rank *P* < .001). Abbreviations: AMI-CS, acute myocardial infarction complicated by cardiogenic shock; CI, confidence interval; MC, mechanical complication; NSTEMI-CS, non-ST-elevation myocardial infarction complicated by cardiogenic shock; OHCA, out-of-hospital cardiac arrest; STEMI-CS, ST-elevation myocardial infarction complicated by cardiogenic shock

Clinical events within 30 days and follow-up durations for each complication are summarized in *Table 2*. Kaplan–Meier estimates of the cumulative incidence of all-cause death at 30 days are shown in *Figure 2*. The 30-day cumulative incidence of death from any cause was 38.7% (95% CI: 37.0–40.2) in the overall AMI-CS population, and varied across subgroups: 37.6% (95% CI: 34.9–40.2) in the Eligible STEMI-CS group, 27.6% (95% CI: 24.2–30.9) in the Non-eligible STEMI-CS group, 51.3% (95% CI: 48.0–54.4) in the OHCA group, 39.8% (95% CI: 32.7–46.2) in the MC group, and 33.3% (95% CI: 28.9–37.5) in the NSTEMI-CS group (log-rank *P* < .001).

**Table 2:** Clinical events within 30 days after Impella implantation

	All *N* = 3975	Eligible STEMI-CS N = 1417 (35.6%)	Non-eligible STEMI-CS N = 820 (20.6%)	OHCA *N* = 992 (25.0%)	MC *N* = 213 (5.4%)	NSTEMI-CS *N* = 533 (13.4%)	*P* value
Death from any cause							
Cumulative incidence at 30 days (95% CI)	38.7 (37.0–40.2)	37.6 (34.9–40.2)	27.6 (24.2–30.9)	51.3 (48.0–54.4)	39.8 (32.7–46.2)	33.3 (28.9–37.5)	<.001
Median follow up period, median (IQR)	23.0 (10.0–30.0)	24.0 (11.0–30.0)	23.5 (14.0–30.0)	18.0 (4.0–30.0)	30.0 (12.0–30.0)	27.0 (13.0–30.0)	<.001
Cardiac death							
Cumulative incidence at 30 days (95% CI)	32.2 (30.6–33.7)	31.2 (28.5–33.7)	22.7 (19.4–25.8)	43.8 (40.4–47)	34.5 (27.4–40.8)	26.6 (22.4–30.5)	<.001
Median follow up period, median (IQR)	23.0 (10.0–30.0)	24.0 (11.0–30.0)	23.5 (14.0–30.0)	18.0 (4.0–30.0)	30.0 (12.0–30.0)	27.0 (13.0–30.0)	<.001
Non-cardiac death							
Cumulative incidence at 30 days (95% CI)	9.5 (8.3–10.6)	9.3 (7.4–11.1)	6.3 (4.4–8.3)	13.1 (10.3–15.8)	8.0 (3.7–12.2)	9.1 (6.2–12.0)	.0014
Median follow up period, median (IQR)	23.0 (10.0–30.0)	24.0 (11.0–30.0)	23.5 (14.0–30.0)	18.0 (4.0–30.0)	30.0 (12.0–30.0)	27.0 (13.0–30.0)	<.001
Major bleeding							
Cumulative incidence at 30 days (95% CI)	26.6 (25.1–28)	28.9 (26.3–31.3)	24.7 (21.5–27.8)	27.5 (24.4–30.5)	17.7 (12.1–22.9)	25.0 (21.0–28.7)	.0019
Median follow up period, median (IQR)	16.0 (2.0–30.0)	15.0 (2.0–30.0)	18.0 (7.0–30.0)	10.0 (1.0–30.0)	22.0 (8.0–30.0)	19.0 (3.0–30.0)	<.001
Impella-related bleeding							
Cumulative incidence at 30 days (95% CI)	13.9 (12.8–15)	15.6 (13.6–17.5)	13.5 (11.1–15.8)	13.8 (11.4–16.1)	6.0 (2.6–9.2)	13.4 (10.4–16.3)	.010
Median follow up period, median (IQR)	19.0 (5.0–30.0)	19.0 (4.0–30.0)	20.0 (10.0–30.0)	15.0 (2.0–30.0)	28.0 (10.0–30.0)	23.0 (6.0–30.0)	<.001
Intracranial bleeding							
Cumulative incidence at 30 days (95% CI)	3.2 (2.6–3.8)	3.3 (2.2–4.4)	2.5 (1.3–3.7)	4.6 (3.0–6.2)	2.5 (.3–4.7)	2.1 (0.8–3.4)	.14
Median follow up period, median (IQR)	23.0 (10.0–30.0)	24.0 (11.0–30.0)	24.0 (14.0–30.0)	19.0 (4.0–30.0)	30.0 (12.0–30.0)	27.0 (12.0–30.0)	<.001
Limb ischaemia							
Cumulative incidence at 30 days (95% CI)	5.8 (5.1–6.6)	6.2 (4.9–7.6)	5.1 (3.6–6.7)	4.5 (3.0–6.0)	4.7 (1.6–7.6)	8.5 (6.0–11)	.016
Median follow up period, median (IQR)	22.0 (9.0–30.0)	22.0 (9.0–30.0)	22.5 (13.0–30.0)	18.0 (4.0–30.0)	29.0 (10.0–30.0)	26.0 (8.0–30.0)	<.001
Worsening renal failure							
Cumulative incidence at 30 days (95% CI)	10.1 (9.1–11.1)	12.2 (10.3–14.0)	9.1 (7.0–11.1)	7.4 (5.5–9.2)	12.0 (7.2–16.5)	10.2 (7.5–12.9)	.003
Median follow up period, median (IQR)	21.0 (7.0–30.0)	20.0 (6.0–30.0)	21.0 (12.0–30.0)	17.0 (3.0–30.0)	27.0 (9.0–30.0)	24.0 (8.0–30.0)	<.001
Ischaemic stroke							
Cumulative incidence at 30 days (95% CI)	5.0 (4.3–5.8)	4.9 (3.6–6.1)	4.5 (3.0–6.1)	5.9 (4.1–7.7)	7 (3.1–10.8)	4.1 (2.2–5.8)	.59
Median follow up period, median (IQR)	22.0 (10.0–30.0)	22.0 (10.0–30.0)	23.0 (13.0–30.0)	17.0 (4.0–30.0)	28.0 (11.0–30.0)	27.0 (11.0–30.0)	<.001
Ventricular arrhythmia							
Cumulative incidence at 30 days (95% CI)	6.0 (5.2–6.8)	6.0 (4.7–7.3)	6.9 (5.0–8.8)	6.3 (4.5–8.0)	1.7 (0–3.7)	6.2 (4.0–8.4)	.13
Median follow up period, median (IQR)	22.0 (9.0–30.0)	23.0 (9.0–30.0)	23.0 (13.0–30.0)	17.5 (3.0–30.0)	30.0 (12.0–30.0)	26.0 (11.0–30.0)	<.001
Sepsis							
Cumulative incidence at 30 days (95% CI)	6.4 (5.5–7.3)	6.3 (4.8–7.7)	6.6 (4.6–8.5)	5.1 (3.4–6.7)	6 (2.0–9.9)	8.6 (5.8–11.2)	.34
Median follow up period, median (IQR)	22.0 (9.0–30.0)	23.0 (10.0–30.0)	23.0 (13.0–30.0)	17.0 (4.0–30.0)	29.0 (11.0–30.0)	26.0 (11.0–30.0)	<.001

Abbreviations: CI, confidence interval; IQR, interquartile range; MC, mechanical complication; NSTEMI-CS, non-ST-segment elevation myocardial infarction complicated by cardiogenic shock; OHCA, out-of-hospital cardiac arrest; STEMI-CS, ST-segment elevation myocardial infarction complicated by cardiogenic shock; VA-ECMO, veno-arterial extracorporeal membrane oxygenation.

Data are presented as cumulative incidence at 30 days with 95% confidence intervals, or median follow-up duration with interquartile ranges. *P* values for comparisons of cumulative incidence across the five groups were calculated using the log-rank test, while those for median follow-up periods were calculated using the Kruskal–Wallis test.

In addition to all-cause mortality, other complications such as cardiac death, non-cardiac death, major bleeding, Impella-related bleeding, limb ischaemia, and renal failure also differed significantly among subgroups.

### Multivariable analysis for 30-day mortality

**Figure 3:**
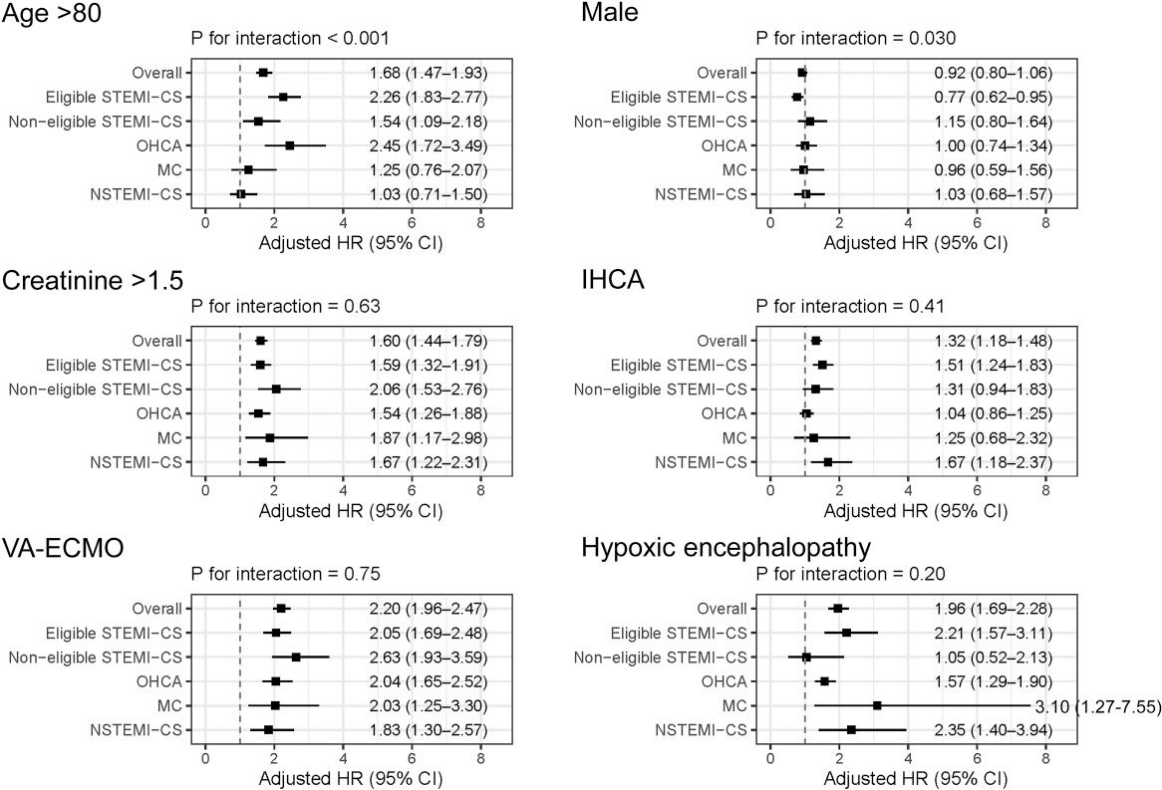

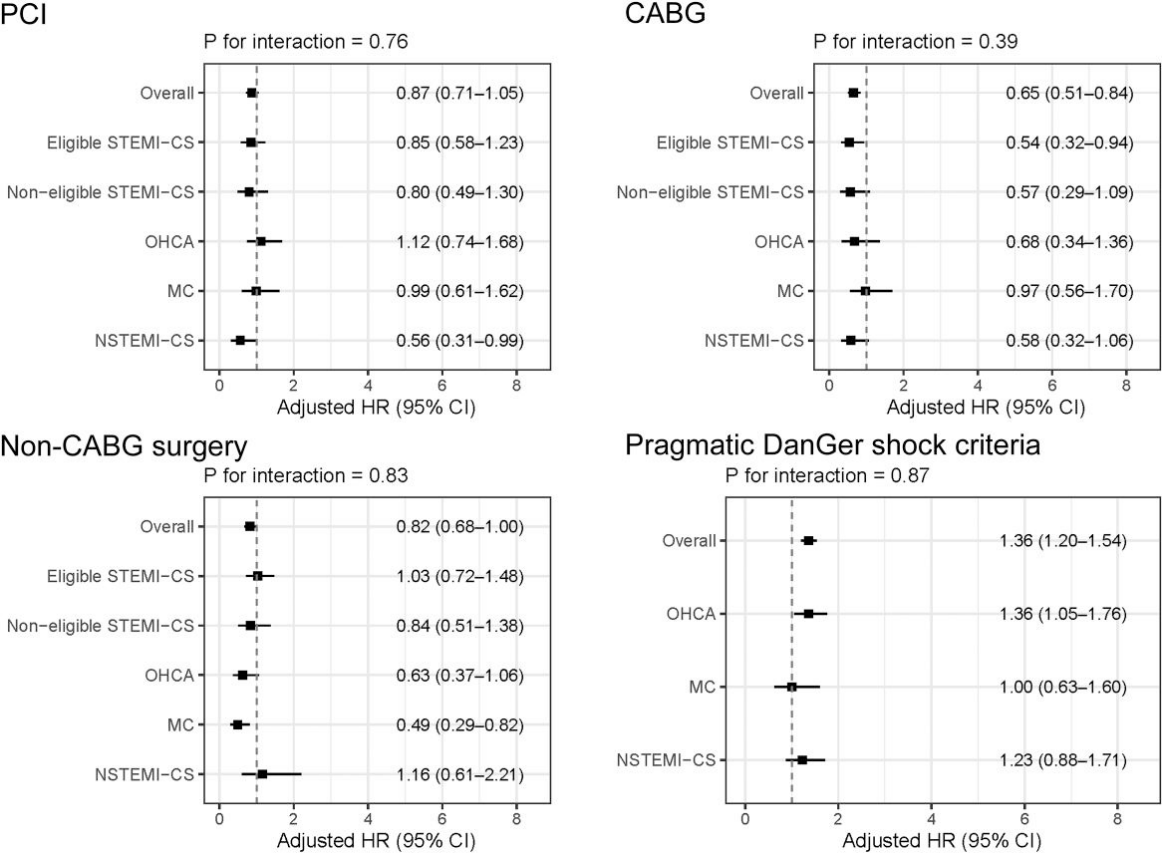
Forest plots of adjusted HRs for 30-day all-cause mortality according to baseline clinical characteristics in AMI-CS patients treated with Impella. Adjusted HRs and 95%CIs are shown for 30-day all-cause mortality according to key baseline characteristics in the overall cohort and across five subgroups of AMI-CS: Eligible STEMI-CS, Non-eligible STEMI-CS, OHCA, MC, and NSTEMI-CS. *(A)* Prognostic risk factors: age > 80, male sex, creatinine > 1.5 mg/dL, IHCA, VA-ECMO, and hypoxic encephalopathy. *(B)* Treatment-related variables: PCI, CABG, non-CABG surgery, and eligibility for the pragmatic DanGer shock trial criteria. *P* for interaction is reported for each characteristic to assess effect modification by subgroup. All adjusted HRs were derived from multivariable models adjusted for the following covariates: age > 80 years, creatinine > 1.5 mg/dL, IHCA, use of VA-ECMO, male sex, hypoxic encephalopathy, PCI, CABG, non-CABG surgery, and the presence of pragmatic DanGer shock criteria. Abbreviations: AMI-CS, acute myocardial infarction complicated by cardiogenic shock; CABG, coronary artery bypass grafting; CI, confidence interval; HR, hazard ratio; IHCA, in-hospital cardiac arrest; MC, mechanical complication; NSTEMI-CS, non-ST-elevation myocardial infarction complicated by cardiogenic shock; OHCA, out-of-hospital cardiac arrest; PCI, percutaneous coronary intervention; STEMI-CS, ST-elevation myocardial infarction complicated by cardiogenic shock

**Figure 4:**
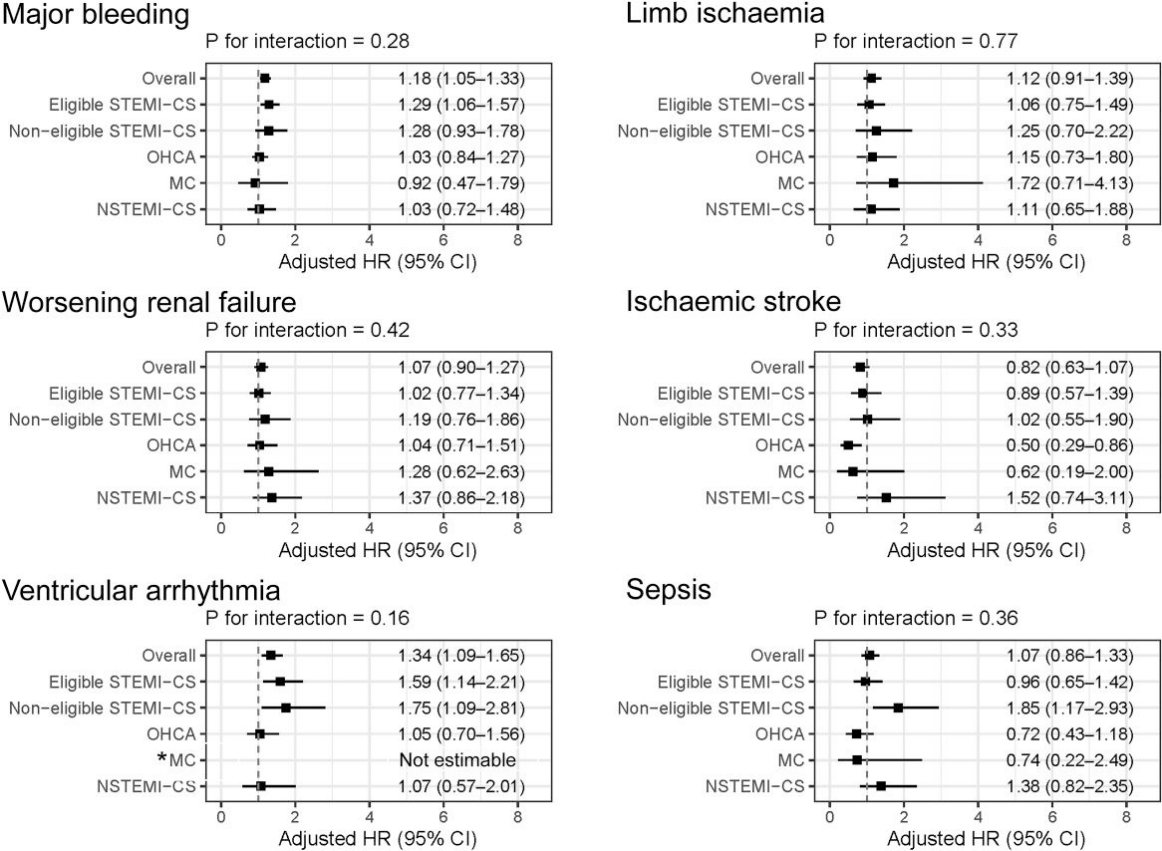
Forest plots of adjusted hazard ratios for 30-day all-cause mortality according to clinical complications in AMI-CS patients treated with Impella. Adjusted HRs and 95%CIs are presented for clinical complications associated with 30-day all-cause mortality in the overall cohort and across five AMI-CS subgroups: Eligible STEMI-CS, Non-eligible STEMI-CS, OHCA, MC, and NSTEMI-CS. *The HR was not estimable for the MC group due to zero deaths among patients with ventricular arrhythmia (*n* = 3). All adjusted HRs were derived from multivariable models adjusted for age > 80 years, male sex, and the occurrence of major complications, including major bleeding, limb ischaemia, worsening renal failure, ischaemic stroke, ventricular arrhythmia, and sepsis. Abbreviations: AMI-CS, acute myocardial infarction complicated by cardiogenic shock; CI, confidence interval; HR, hazard ratio; MC, mechanical complication; NSTEMI-CS, non-ST- elevation myocardial infarction complicated by cardiogenic shock; OHCA, out-of-hospital cardiac arrest; STEMI-CS, ST-elevation myocardial infarction complicated by cardiogenic shock

Multivariable analyses for 30-day mortality are presented as forest plots (*Figs 3* and *4*), showing adjusted HRs with 95% CIs in the overall AMI-CS population and each clinical subgroup. Among clinical variables (*Figure 3A*), age > 80 years was significantly associated with higher mortality in the overall population (adjusted HR 1.68, 95% CI: 1.47–1.93), with the effect most pronounced in the OHCA and Eligible STEMI-CS groups. Notably, a significant interaction was observed across subgroups (*P* for interaction < .001), suggesting heterogeneous prognostic impact. Male sex was associated with reduced mortality in the Eligible STEMI-CS group (adjusted HR 0.77, 95% CI: 0.62–0.95), with a significant interaction (*P* = .030). In contrast, variables such as elevated creatinine (>1.5 mg/dL), IHCA, and VA-ECMO use were consistently associated with increased risk, without significant subgroup interactions (*P* for interaction >0.4). Hypoxic encephalopathy showed the highest hazard in the MC group (adjusted HR 3.10, 95% CI: 1.27–7.55), though the interaction was not statistically significant (*P* = .20).

In terms of procedural and treatment-related factors (*Figure 3B*), PCI was associated with a trend towards lower mortality in the overall population (adjusted HR 0.87, 95% CI: 0.71–1.05), and this association reached statistical significance in the NSTEMI-CS group (adjusted HR 0.56, 95% CI: 0.31–0.99), although no significant subgroup interaction was observed (*P* for interaction = 0.76). CABG was significantly associated with lower mortality in the overall population (adjusted HR 0.65, 95% CI: 0.51–0.84), without evidence of subgroup heterogeneity (*P* = .39). Non-CABG surgery was associated with reduced mortality in the MC group (adjusted HR 0.49, 95% CI: 0.29–0.82), but the interaction across subgroups was not significant (*P* = .83). The presence of the pragmatic DanGer shock criteria was independently associated with increased mortality in the overall cohort (adjusted HR 1.36, 95% CI: 1.20–1.54), with no significant interaction across subgroups (*P* for interaction = .87).

Complications occurring within 30 days were analysed in *Figure 4*. Major bleeding (adjusted HR 1.18, 95% CI: 1.05–1.33), and ventricular arrhythmia (adjusted HR 1.34, 95% CI: 1.09–1.65) were associated with increased mortality in the overall cohort. However, none of the complication-related variables showed significant interaction with subgroup classification, suggesting consistent prognostic impact across groups. Importantly, in the Non-eligible STEMI-CS group, both ventricular arrhythmia (adjusted HR 1.75, 95% CI: 1.09–2.81) and sepsis (adjusted HR 1.85, 95% CI: 1.17–2.93) were significantly associated with increased 30-day mortality.

To assess the predictive performance of the multivariable models, concordance indices (*c*-statistics) were calculated (see online supplementary data online, *Table S2*). Model 1 demonstrated acceptable discrimination for 30-day mortality in the overall population (*c*-index = 0.70) and across all subgroups (range: 0.64–0.71). By contrast, Model 2, which incorporated complications, showed lower discriminatory performance (*c*-index = 0.54–0.61), suggesting that baseline clinical characteristics were more useful for risk stratification than complications alone.

Multicollinearity among covariates was evaluated using VIFs and condition indices (see online supplementary data online, *Table S3*). In all models and subgroups, VIF values were consistently below 2.0 and condition indices were well below the commonly accepted threshold of 30, indicating no significant multicollinearity.

### Subgroup analysis by timing of VA-ECMO in Impella-treated eligible STEMI-CS patients

Among 1417 patients classified as Eligible STEMI-CS, 1062 (74.9%) received Impella first, while 355 (25.1%) received VA-ECMO prior to Impella (see online supplementary data online, *Table S4*). VA-ECMO was subsequently required in 22.5% of the Impella-first group. Compared with the VA-ECMO-first group, the Impella-first group had more favourable baseline characteristics, including lower rates of IHCA and hypoxic encephalopathy, higher systolic blood pressure, and lower lactate levels.

Cumulative 30-day mortality was significantly lower in the Impella-first group (32.6% vs 52.1%), while ventricular arrhythmia and sepsis occurred at similar rates and all other clinical events were more frequent in this group (see online supplementary data online, *Table S5*).

### Subgroup analysis by pre-hospital ROSC status in OHCA patients treated with Impella

Compared with patients without ROSC, those with pre-hospital ROSC were older and had more comorbidities, including diabetes and prior heart failure. They also presented with higher systolic blood pressure and better LVEF (see online supplementary data online, *Table S6*).

VA-ECMO was used less frequently in the ROSC group (55.0% vs 76.6%), and was initiated prior to Impella in 82.9% vs 98.2%, respectively. Hypoxic encephalopathy was significantly less frequent in the ROSC group (22.9% vs 31.6%).

Among 992 OHCA patients, the most common underlying cause was STEMI-CS (73.9%), followed by NSTEMI-CS (24.4%) and MC (1.7%). Among the 638 patients with pre-hospital ROSC, STEMI-CS accounted for 496 cases (77.7%), which represented 50.0% of the entire OHCA cohort (496/992).

Despite having more comorbidities, patients with pre-hospital ROSC showed lower cumulative 30-day mortality (44.5% vs 63.4%) and cardiac death (37.0% vs 56.0%) compared with those without ROSC (see online supplementary data online, *Table S7*).

### Subgroup analysis by age in patients with mechanical complications treated with Impella

Older patients with MC (age > 80 years) exhibited a significantly higher proportion of female sex and lower rates of current smoking and non-CABG surgery. Use of IABP and VA-ECMO was similar between age groups (see online supplementary data online, *Table S8*).

Cumulative 30-day mortality was higher in older patients (52.2% vs 35.5%; *P* = .041), mainly driven by more cardiac deaths (51.2% vs 28.5%; *P* = .005). Other complications occurred at similar rates across age groups (see online supplementary data online, *Table S9*).

## Discussion

### Major findings

Among the 3975 AMI-CS patients, 1417 (35.6%) were classified as Eligible STEMI-CS based on a pragmatic adaptation of the DanGer shock trial criteria. The remaining 2558 patients (64.4%) did not meet these criteria and were stratified into four subgroups: Non-eligible STEMI-CS (20.6%), OHCA (25.0%), MCs (5.4%), and NSTEMI-CS (13.4%). Cumulative incidence of 30-day mortality was 37.6% in the Eligible STEMI-CS group, while varying across the other subgroups—ranging from 27.6% in Non-eligible STEMI-CS to 51.3% in OHCA. Multivariable analyses revealed that clinical risk factors and complications—including renal dysfunction, IHCA, VA-ECMO use, hypoxic encephalopathy, major bleeding, and ventricular arrhythmia—were associated with increased mortality, with no significant interaction observed across subgroups (*Structured Graphical Abstract*).

These findings delineate the real-world use and outcomes of Impella in a broad AMI-CS population—including patients outside the original DanGer shock trial criteria—and underscore the importance of clinical and procedural risk stratification in guiding patient selection.

### MCS for AMI-CS

In Japan, recent data from the J-PCI registry (2019–21) involving 12 171 AMI-CS patients undergoing PCI showed that IABP alone was the most commonly used MCS (60.0%), followed by VA-ECMO (34.9%) and Impella alone (5.1%).^15^ Over time, overall MCS use increased, with IABP decreasing (from 63.5% to 58.3%) and Impella increasing. Notably, the use of VA-ECMO combined with Impella (ECPELLA) rose from 4.2% to 17.0%.

In contrast, a contemporary North American CICU registry (2019–23) reported different trends among STEMI-CS patients meeting DanGer shock-like eligibility: IABP was used in 44.1%, Impella in 21.6%, and VA-ECMO in 9.8%.^16^ These differences reflect regional variation in device availability and practice patterns.

The present J-PVAD registry exclusively included certified Impella institutions, where all AMI-CS patients received Impella. Consequently, IABP was used in only 14.3%. VA-ECMO was more common (43.9%), likely reflecting the severity of illness requiring both devices in advanced centres. Importantly, in this cohort, 1231 AMI-CS patients (31.0% of 3975) received VA-ECMO before Impella initiation, indicating that Impella was not the first-line MCS in these cases.

### Eligible STEMI-CS

In the present study, 35.6% of AMI-CS patients met the pragmatic DanGer shock criteria for STEMI-CS, with a 30-day all-cause mortality rate of 37.6%. This proportion is notably higher than that reported in European observational studies, where DanGer shock-like eligibility was fulfilled by only 24.4%^6^ to 28.4%^17^ of patients. Conversely, the 30-day mortality observed in the present study was lower than that reported in these studies (30-day mortality of 64.8%^6^ and 180-day mortality of 62.5%,^17^ respectively). Moreover, male sex was independently associated with lower 30-day mortality specifically in the Eligible STEMI-CS group—a trend consistent with findings from the DanGer shock trial, where Impella showed greater mortality reduction in male patients.^5^ This suggests that sex-based differences may influence treatment response in selected STEMI-CS populations and merit further investigation.

An important distinction is that, in the Eligible STEMI-CS group, 594 patients (41.9%) received VA-ECMO during Impella support. Among the 1417 patients classified as Eligible STEMI-CS, only a subset received Impella as the first-line MCS; notably, 355 patients (25.1%) underwent VA-ECMO before Impella initiation. In contrast, in the Impella arm of the DanGer shock trial, Impella was used as the first-line MCS, with subsequent VA-ECMO use in only 11.7% of patients. This highlights that, unlike the DanGer shock trial where Impella was deployed upfront, many patients in the present cohort received it as part of an escalation strategy. These differences in MCS sequencing, along with heterogeneity in baseline characteristics, limit the direct comparability of outcomes between the two cohorts.

To approximate the original Impella arm more closely, a subgroup analysis was performed (see online supplementary data online, *Tables S4* and *S5*). In this subgroup, the Impella-first group showed lower 30-day mortality (32.6% vs 52.1%) and required VA-ECMO less frequently (22.5%) than the VA-ECMO-first group. These findings suggested that the survival benefit associated with Impella observed in the DanGer shock trial may also be reproducible in real-world STEMI-CS patients who meet the pragmatic eligibility criteria, although outcomes may differ depending on whether Impella or VA-ECMO is used as the first-line MCS, warranting further investigation.

### AMI-CS patients outside the eligible STEMI-CS group

Previous observational studies, including those by Mangner *et al*. and Schrage *et al*., analysed patients who did not meet the DanGer shock criteria as a single group.^6,17^ These studies reported poor outcomes, with 30-day mortality of 60.3% in Schrage *et al*. and 180-day mortality of 72.0% in Mangner *et al*., but their approach lacked clinical granularity and failed to reflect the diversity of real-world AMI-CS presentations.

In contrast, the present study stratified the non-eligible population—those outside the Eligible STEMI-CS group—into four clinically meaningful subgroups: OHCA, MC, NSTEMI-CS, and non-eligible STEMI-CS. This classification was based on typical clinical presentations encountered in practice and revealed substantial differences in 30-day mortality, ranging from 27.6% in non-eligible STEMI-CS to 51.3% in OHCA. These findings underscore the heterogeneity of AMI-CS patients receiving Impella and highlight the importance of tailored risk assessment.

By distinguishing subgroups that are often lumped together, this approach offers a more nuanced and clinically relevant framework for evaluating patient outcomes and the potential role of Impella in populations not represented in the DanGer shock trial.

### Non-eligible STEMI-CS

This group, defined by the absence of OHCA and MC and not meeting the pragmatic DanGer shock criteria, showed higher systolic blood pressure, lower catecholamine use, higher LVEF, and lower lactate levels compared with other groups—features consistent with SCAI shock stage B.^18^ While no randomized trial has evaluated the efficacy of Impella in this population, it had the lowest 30-day mortality (27.6%) in the present study.

Pre-clinical and observational studies suggest that Impella may provide myocardial protection through left ventricular unloading. In an animal model, Saku *et al*. demonstrated that full left ventricular unloading reduced infarct size in left anterior descending artery (LAD)-related AMI.^19^ In a clinical study, Fukamachi *et al*. reported that Impella preserved remote (non-infarcted) myocardium in LAD-related AMI, compared with IABP.^20^ The ongoing STEMI-DTU trial is expected to clarify whether such myocardial protection translates into improved clinical outcomes in humans.^21^

Despite the overall favourable prognosis in this lower-risk group, mortality increased significantly in the presence of established risk factors such as age >80 years, renal dysfunction, and VA-ECMO use. Additionally, complications such as ventricular arrhythmias and sepsis were also associated with increased mortality. These findings underscore the importance of carefully weighing the potential cardioprotective benefits of left ventricular unloading with Impella against the risks of comorbidities and complications in this lower-risk subgroup.

### OHCA

One in four AMI-CS patients treated with Impella (25.0%) presented with OHCA. Among them, 62.7% required VA-ECMO, with nearly 90% receiving it prior to Impella. This group had the highest 30-day mortality (51.3%), which increased further to 63.4% among patients without pre-hospital ROSC. Neurological injury was a key determinant of outcome.^22^ Hypoxic encephalopathy occurred in 26.0% of OHCA patients and was independently associated with increased mortality (adjusted HR 1.57, 95% CI: 1.29–1.90).

Among the 992 OHCA patients, STEMI-CS was the most common underlying cause (73.9%). In those with pre-hospital ROSC, STEMI-CS accounted for 496 patients (77.7%), representing 50.0% of the entire OHCA cohort. While neurological status was not formally recorded, this subgroup may include patients who would have met DanGer shock inclusion criteria had coma been excluded. However, the relatively high incidence of hypoxic encephalopathy highlights the need for caution when considering eligibility.

Early risk stratification is essential in this complex population. The MIRACLE2 score has been proposed to predict neurological outcomes after cardiac arrest of presumed cardiac origin, while the J-PVAD score offers a practical means of estimating early mortality in patients receiving VA-ECMO plus Impella.^9,23^ These tools may help identify OHCA patients more likely to benefit from Impella by facilitating timely evaluation of hypoxic encephalopathy and baseline risk, though further validation is needed.

### MC

MCs occur in ∼0.2% of AMI cases and carry persistently high mortality despite growing MCS use. Impella utilization in MC remains limited (0%–27.3%), and robust evidence is lacking.^24,25^

In the present study, 74.2% of MC patients underwent non-CABG surgery, with a 30-day mortality of 39.8%, slightly lower than previously reported rates (43.9%–66.4%).^24,25^ Subgroup analysis showed that patients aged >80 years were more often female and had higher cumulative 30-day mortality, yet age >80 was not independently associated with death and showed significant interaction, suggesting age alone may not drive poor prognosis. Instead, 30-day mortality was more strongly linked to hemodynamic compromise—creatinine >1.5 mg/dL, VA-ECMO use, and hypoxic encephalopathy. Notably, non-CABG surgery was associated with reduced mortality (adjusted HR 0.49, 95% CI: 0.29–0.82).

Previous studies suggest Impella may provide better hemodynamic support than IABP in ventricular septal rupture, with case reports supporting ECPELLA as a bridge to surgery.^26,27^ Collectively, these findings indicate that managing MC-related hemodynamic collapse with timely MCS and surgical intervention may be critical to improving outcomes.

### NSTEMI-CS

NSTEMI-CS was not included in the DanGer shock trial, yet its prevalence is increasing in contemporary practice. A Danish registry reported a decline in the proportion of STEMI-CS from 81% in 2010% to 61.2% in 2017, with NSTEMI-CS demonstrating higher 30-day mortality than STEMI-CS (57.7% vs 46.1%).^2^ Furthermore, a recent analysis linking early Impella use to improved survival included only 18% NSTEMI-CS patients, underscoring the limited evidence available for this subgroup.^7^

In the present study, among NSTEMI-CS patients without OHCA or MCs, the 30-day mortality was 33.3%, which compares favourably—at least numerically—with the 30-day mortality of 57.7% reported in the Danish registry.^2^ This group, characterized by the oldest median age (75 years), also had the highest prevalence of standard modifiable cardiovascular risk factors—hypertension, dyslipidaemia, and diabetes—along with a history of heart failure, CKD, and haemodialysis, suggesting a population with complex or multivessel coronary disease and a correspondingly poor prognosis.^28,29^ Importantly, in this high-risk group, both PCI (adjusted HR 0.56, 95% CI: 0.31–0.99) and CABG (adjusted HR 0.58, 95% CI: 0.32–1.06) were associated with lower 30-day mortality. These findings imply that selected NSTEMI-CS patients may benefit from revascularisation with Impella support, warranting further investigation.

### Limitations

This study has several limitations. First, it was based on a retrospective analysis of the J-PVAD registry, and the decision to use Impella, as well as the timing of its initiation, was left to the discretion of the treating physician. This may have introduced selection bias.

Second, when compared with the DanGer shock trial, the present definition of Eligible STEMI-CS differs in three important respects: (i) although the DanGer shock trial applied 13 exclusion criteria, the present analysis explicitly accounted for only 6: shock duration time >24 h, other causes of shock, MCs, OHCA, severe peripheral arterial disease precluding Impella, and aortic abnormalities precluding Impella. The remaining seven exclusion conditions—such as severe right ventricular failure, severe aortic valve disease, mechanical aortic valve prosthesis, left ventricular thrombus, infective endocarditis, life expectancy <1 year due to comorbidities, and mental disorder or language barrier—were not assessed due to limitations in the registry data (see online supplementary data online, *Table S1*). However, these conditions typically preclude the use of Impella in real-world clinical practice, particularly at experienced centres, and are thus likely underrepresented in the registry population. Still, the possibility remains that some patients meeting one or more of these exclusion criteria may have been included in the Eligible STEMI-CS group. (ii) All OHCA patients were excluded from the Eligible STEMI-CS group, whereas the DanGer shock trial excluded only those with persistent coma (Glasgow Coma Scale [GCS] < 8). Because GCS scores following ROSC are not available in the J-PVAD registry, it is not possible to distinguish between comatose and non-comatose patients post-ROSC. A subgroup analysis showed that among OHCA patients with pre-hospital ROSC, 77.7% had STEMI and 30-day mortality was 44.5%—numerically higher than that of the Eligible STEMI-CS group (37.6%). Furthermore, the frequency of hypoxic encephalopathy in this group remained higher (22.9% vs 4.3%), justifying their retention in the OHCA category. Nevertheless, some patients who would have qualified for the DanGer shock trial based on neurological status may have been misclassified. (iii) In addition, approximately one-quarter of Eligible STEMI-CS patients received VA-ECMO prior to Impella initiation, representing an escalation strategy rather than first-line Impella use. Although subgroup analyses were conducted to address this heterogeneity, comparisons with the DanGer shock trial—where Impella was consistently used as the initial MCS—should be interpreted with caution.

Third, neurological outcomes were not assessed, which is particularly relevant in the OHCA group given the high rate of hypoxic encephalopathy; inclusion of standardized neurological measures in future registry updates would enable more accurate prognostic assessment.

Fourth, inter-centre variability in operator experience and treatment protocols may have influenced outcomes.

Finally, cost-related data were not collected in the J-PVAD registry, precluding formal cost-effectiveness analysis. Future studies should incorporate economic endpoints to inform resource allocation and policy decisions.

## Conclusion

In this nationwide registry of Japanese AMI-CS patients treated with Impella, 35.6% were classified as Eligible STEMI-CS, with a 30-day mortality rate of 37.6%. Among STEMI-CS patients not meeting the pragmatic DanGer shock criteria—likely reflecting a lower-risk subgroup similar to SCAI shock stage B—mortality was lower but increased in the presence of established risk factors or complications. Mortality remained high in patients with OHCA, particularly among those without pre-hospital ROSC, while outcomes in MCs and NSTEMI-CS were within a reasonable range, suggesting a possible benefit of Impella in selected cases. These findings underscore the importance of careful patient selection and highlight the need for further research in real-world AMI-CS populations beyond current trial frameworks.

## Supplementary Material

ehaf787_Supplementary_Data

## Declarations

### Data Availability

The deidentified participant data will not be shared.
