# Supplementary material for: DanGer shock criteria and outcomes in acute myocardial infarction-related cardiogenic shock treated with Impella: the J-PVAD registry
Source: Eur Heart J. 2025 Oct 13;47(4):472–86. doi: 10.1093/eurheartj/ehaf787 (PMC12831186; doi:10.1093/eurheartj/ehaf787)
Supplement: ehaf787_Supplementary_Data [file ehaf787_supplementary_data.docx]

**Supplementary material**

**DanGer shock criteria and outcomes in acute myocardial infarction-related cardiogenic shock treated with Impella: the J-PVAD registry**

Riku Arai, MD, Keisuke Kojima, MD, Daisuke Fukamachi, MD, Yasuo Okumura, MD, J-PVAD investigators

^*^**Corresponding author:** Riku Arai, MD

Division of Cardiology, Department of Medicine, Nihon University School of Medicine, Tokyo, Japan

Oyaguchikamicho 30-1, Itabashi-ku, Tokyo 1738610

Tel: +81-3-3972-8111

E-mail: riku.arai@icloud.com

**Supplementary Tables**

**Table S1: Treatment of DanGer shock Exclusion Criteria in the J-PVAD Registry**

**Table S2. Concordance Index (c-statistics) for 30-Day Mortality in Multivariate Cox Models**

**Table S3. Evaluation of Multicollinearity Using Variance Inflation Factor and Condition Index Across Cox Regression Models**

**Table S4. Baseline characteristics and procedure details in patients with eligible STEMI-CS, categorized by whether Impella or VA-ECMO was introduced first**

**Table S5. Clinical events within 30 days after Impella implantation in patients with Eligible STEMI-CS, categorized by whether Impella or VA-ECMO was initiated first**

**Table S6. Baseline characteristics and procedure details in OHCA patients, categorized by pre-hospital ROSC status**

**Table S7. Clinical events within 30 days after Impella implantation in patients with OHCA, categorized by pre-hospital ROSC status**

**Table S8. Baseline characteristics, and procedure details in patients with mechanical complications, particularly focusing on age stratification**

**Table S9. Clinical events within 30 days after Impella implantation in patients with mechanical complications**, **particularly focusing on age stratification**

**Table S1. Treatment of DanGer shock Exclusion Criteria in the J-PVAD Registry**

| No. | DanGer shock Exclusion Criterion | J-PVAD Variable Available | Operationalization | Excluded from the 3,975 Study Cases (n) | Potentially Included in Eligible STEMI-CS Due to Non-application of Exclusion Criteria |
| --- | --- | --- | --- | --- | --- |
| 1 | Shock duration >24 hours | Yes | Shock onset to first Impella support >24 h | 230 in STEMI-CS | None |
| 2 | Other causes of shock | Yes | All 3,975 cases were AMI-related cardiogenic shock | Automatically excluded by design | None |
| 3 | Shock due to mechanical complication | Yes | Explicitly recorded as mechanical complication | 213 | None |
| 4 | OHCA with GCS <8 after ROSC | Yes | Based on OHCA status; GCS not available | 992 | None; Due to the absence of GCS data, some of the 638 OHCA cases with ROSC may have been inappropriately excluded; a subgroup analysis was conducted. |
| 5 | Severe right ventricular failure | No | Not available | 0 | Possible |
| 6 | Severe aortic valve regurgitation/stenosis | No | Not available | 0 | Possible |
| 7 | Severe peripheral arterial disease precluding Impella | No | Not available | Not applicable; all 3,975 cases had successful Impella implantation.  The 53 cases with unsuccessful device implantation were excluded from the registry dataset. | None |
| 8 | Aortic abnormalities precluding Impella | No | Not available | Not applicable; all 3,975 cases had successful Impella implantation.  The 53 cases with unsuccessful device implantation were excluded from the registry dataset. | None |
| 9 | Mechanical aortic valve prosthesis | No | Not available | 0 | Possible (contraindicated for Impella; likely excluded in practice) |
| 10 | Left ventricular thrombus | No | Not available | 0 | Possible |
| 11 | Infective endocarditis | No | Not available | 0 | Possible |
| 12 | Life expectancy <1 year due to comorbidities | No | Not available | 0 | Possible |
| 13 | Mental disorder or language barrier | No | Not available | 0 | Possible |

Abbreviations: AMI, acute myocardial infarction; GCS, Glasgow Coma Scale; J-PVAD, Japanese Percutaneous Ventricular Assist Device; OHCA, out-of-hospital cardiac arrest; ROSC, return of spontaneous circulation; STEMI-CS, ST-segment elevation myocardial infarction complicated by cardiogenic shock; VA-ECMO, veno-arterial extracorporeal membrane oxygenation

**Table S2. Concordance Index (c-statistics) for 30-Day Mortality in Multivariate Cox Models**

|  | Overall | Eligible STEMI-CS | Non-eligible STEMI-CS | OHCA | MC | NSTEMI-CS |
| --- | --- | --- | --- | --- | --- | --- |
| Model 1 | 0.70 | 0.70 | 0.71 | 0.64 | 0.67 | 0.69 |
| Model 2 | 0.54 | 0.61 | 0.59 | 0.53 | 0.58 | 0.55 |

Abbreviations: MC, mechanical complication; NSTEMI-CS, non–ST-segment elevation myocardial infarction complicated by cardiogenic shock; OHCA, out-of-hospital cardiac arrest; STEMI-CS, ST-segment elevation myocardial infarction complicated by cardiogenic shock

**Table S3. Evaluation of Multicollinearity Using Variance Inflation Factor and Condition Index Across Cox Regression Models**

| Model 1 | Overall | | Eligible STEMI-CS | | Non-eligible STEMI-CS | | OHCA | | MC | | NSTEMI-CS | |
| --- | --- | --- | --- | --- | --- | --- | --- | --- | --- | --- | --- | --- |
| Variables | VIF | Condition index | VIF | Condition index | VIF | Condition index | VIF | Condition index | VIF | Condition index | VIF | Condition index |
| Age >80 | 1.07 | 1.00 | 1.07 | 1.00 | 1.07 | 1.00 | 1.05 | 1.00 | 1.13 | 1.00 | 1.05 | 1.00 |
| Creatinine >1.5 mg/dL | 1.02 | 3.04 | 1.02 | 2.73 | 1.05 | 2.71 | 1.04 | 3.73 | 1.05 | 2.49 | 1.03 | 2.83 |
| IHCA | 1.17 | 3.38 | 1.17 | 3.20 | 1.19 | 2.97 | 1.05 | 3.89 | 1.23 | 2.74 | 1.21 | 3.22 |
| VA-ECMO use | 1.15 | 3.73 | 1.13 | 3.50 | 1.15 | 3.27 | 1.06 | 4.18 | 1.08 | 2.80 | 1.19 | 3.33 |
| Male | 1.06 | 3.87 | 1.05 | 3.58 | 1.06 | 3.81 | 1.04 | 4.33 | 1.09 | 2.91 | 1.08 | 3.52 |
| Hypoxic encephalopathy | 1.06 | 4.11 | 1.06 | 4.71 | 1.06 | 4.13 | 1.03 | 5.30 | 1.11 | 3.21 | 1.08 | 3.69 |
| PCI | 1.28 | 4.77 | 1.10 | 5.75 | 1.21 | 4.75 | 1.23 | 6.36 | 1.11 | 3.61 | 1.46 | 4.21 |
| CABG | 1.20 | 5.37 | 1.11 | 7.12 | 1.13 | 5.50 | 1.23 | 7.78 | 1.11 | 3.77 | 1.53 | 4.87 |
| Non-CABG surgery | 1.18 | 5.75 | 1.06 | 7.45 | 1.11 | 9.16 | 1.06 | 9.43 | 1.24 | 4.69 | 1.13 | 6.67 |
| Pragmatic DanGer shock criteria status | 1.07 | 6.73 | - | - | - | - | 1.06 | 11.19 | 1.10 | 8.53 | 1.05 | 7.70 |

| Model 2 | Overall | | Eligible STEMI-CS | | Non-eligible STEMI-CS | | OHCA | | MC | | NSTEMI-CS | |
| --- | --- | --- | --- | --- | --- | --- | --- | --- | --- | --- | --- | --- |
| Variables | VIF | Condition index | VIF | Condition index | VIF | Condition index | VIF | Condition index | VIF | Condition index | VIF | Condition index |
| Age >80 | 1.04 | 1.00 | 1.05 | 1.00 | 1.04 | 1.00 | 1.03 | 1.00 | 1.07 | 1.00 | 1.01 | 1.00 |
| Male | 1.04 | 2.19 | 1.04 | 2.11 | 1.07 | 2.15 | 1.03 | 2.31 | 1.05 | 1.71 | 1.01 | 2.16 |
| Major bleeding | 1.06 | 2.47 | 1.07 | 2.37 | 1.09 | 2.37 | 1.05 | 3.84 | 1.14 | 2.05 | 1.02 | 2.34 |
| Limb ischaemia | 1.03 | 3.28 | 1.04 | 3.08 | 1.03 | 3.32 | 1.06 | 4.22 | 1.15 | 2.68 | 1.01 | 3.20 |
| Worsening renal failure | 1.09 | 4.32 | 1.11 | 4.13 | 1.07 | 4.06 | 1.17 | 4.66 | 1.21 | 3.48 | 1.07 | 3.66 |
| Ischaemic stroke | 1.02 | 4.43 | 1.02 | 4.44 | 1.04 | 4.17 | 1.02 | 5.05 | 1.04 | 4.21 | 1.01 | 3.92 |
| Ventricular arrhythmia | 1.05 | 4.54 | 1.06 | 4.68 | 1.07 | 4.40 | 1.04 | 5.57 | 1.05 | 4.61 | 1.04 | 4.52 |
| Sepsis | 1.05 | 4.91 | 1.06 | 5.07 | 1.08 | 4.96 | 1.08 | 5.60 | 1.08 | 7.12 | 1.05 | 5.29 |

Abbreviations: MC, mechanical complication; NSTEMI-CS, non–ST-segment elevation myocardial infarction complicated by cardiogenic shock; OHCA, out-of-hospital cardiac arrest; STEMI-CS, ST-segment elevation myocardial infarction complicated by cardiogenic shock, VIF, Variance Inflation Factor

**Table S4. Baseline characteristics and procedure details in patients with eligible STEMI-CS, categorized by whether Impella or VA-ECMO was introduced first**

|  | Eligible STEMI-CS all  N = 1417 | Impella-first  N = 1062 (74.9%) | VA-ECMO-first  N = 355 (25.1%) | P value |
| --- | --- | --- | --- | --- |
| Age, median (IQR) | 72.0 (64.0-79.0) | 73.0 (65.0-79.0) | 71.0 (60.0-76.0) | <0.001 |
| Age >80, n (%) | 265/1,417(18.7) | 216 (20.3) | 49 (13.8) | 0.006 |
| Male, n (%) | 1,133/1,417 (80.0) | 847 (79.8) | 286 (80.6) | 0.74 |
| Body mass index, kg/m2, median (IQR) | 23.3 (20.8-25.8) | 23.1 (20.7-25.5) | 23.8 (21.6-26.5) | <0.001 |
| Hypertension, n (%) | 902/1,417 (63.7) | 687 (64.7) | 215 (60.6) | 0.16 |
| Dyslipidemia, n (%) | 702/1,417 (49.5) | 532 (50.1) | 170 (47.9) | 0.47 |
| Diabetes mellitus, n (%) | 610/1,417 (43.0) | 465 (43.8) | 145 (40.8) | 0.33 |
| Current smoking, n (%) | 446/1,417 (31.5) | 346 (32.6) | 100 (28.2) | 0.12 |
| Prior myocardial infarction, n (%) | 237/1,417 (16.7) | 184 (17.3) | 53 (14.9) | 0.30 |
| History of heart failure, n (%) | 168/1,417 (11.9) | 125 (11.8) | 43 (12.1) | 0.86 |
| Chronic kidney disease, n (%) | 397/1,417 (28.0) | 308 (29.0) | 89 (25.1) | 0.15 |
| Haemodialysis, n (%) | 38/1,417 (2.7) | 23 (2.2) | 15 (4.2) | 0.04 |
| Prior stroke/TIA, n (%) | 107/1,417 (7.6) | 81 (7.6) | 26 (7.3) | 0.85 |
| IHCA, n (%) | 510/1,417 (36.0) | 257 (24.2) | 253 (71.3) | <0.001 |
| Systolic blood pressure, mmHg, median (IQR) | 84.0 (68.0-98.0) | 85.0 (70.0-98.0) | 80.0 (50.0-99.0) | <0.001 |
| Systolic blood pressure <100, mmHg, n (%) | 1,095/1,417(77.3) | 827 (77.9) | 268 (75.5) | 0.35 |
| Diastolic blood pressure, mmHg, median (IQR) | 55.0 (41.0-68.0) | 55.0 (43.0-67.0) | 56.0 (27.0-70.0) | 0.11 |
| Heart rate, bpm, median (IQR) | 90.0 (68.0-110.0) | 91.0 (71.0-110.0) | 84.0 (61.0-109.0) | <0.001 |
| Creatinine, mg/dL, median (IQR) | 1.2 (1.0-1.6) | 1.2 (1.0-1.6) | 1.2 (0.9-1.6) | 0.63 |
| Creatinine >1.5 mg/dL, n (%) | 392/1,397 (28.1) | 291 (27.8) | 101 (28.9) | 0.67 |
| Albumin, g/dL, median (IQR) | 3.6 (3.2-4.0) | 3.7 (3.3-4.0) | 3.5 (3.0-3.9) | <0.001 |
| Lactate, mmol/L, median (IQR) | 6.2 (4.2-9.7) | 5.8 (3.9-8.8) | 8.0 (4.9-11.8) | <0.001 |
| Lactate ≥2.5 mmol/L, n (%) | 1417 (100) | 1062 (100) | 355 (100) | - |
| LVEF, %, median (IQR) | 25.0 (20.0-30.0) | 26.0 (20.0-30.0) | 20.0 (15.0-30.0) | <0.001 |
| LVEF <45%, n (%) | 1417 (100) | 1062 (100) | 355 (100) | - |
| Catecholamine use at initiation of Impella support |  |  |  |  |
| Epinephrine, n (%) | 225/1,417 (15.9) | 112 (10.5) | 113 (31.8) | <0.001 |
| Dobutamine, n (%) | 393/1,417 (27.7) | 296 (27.9) | 97 (27.3) | 0.84 |
| Norepinephrine, n (%) | 821/1,417 (57.9) | 634 (59.7) | 187 (52.7) | 0.02 |
| Dopamine, n (%) | 137/1,417 (9.7) | 103 (9.7) | 34 (9.6) | 0.95 |
| Any catecholamine, n (%) | 1,170/1,417 (82.6) | 849 (79.9) | 321 (90.4) | <0.001 |
| Use of pulmonary artery catheter, n (%) | 905/1,417 (63.9) | 675 (63.6) | 230 (64.8) | 0.68 |
| Hypoxic encephalopathy, n (%) | 61/1,417 (4.3) | 27 (2.5) | 34 (9.6) | <0.001 |
| PCI, n (%) | 1,334/1,417 (94.1) | 1,003 (94.4) | 331 (93.2) | 0.40 |
| CABG, n (%) | 69/1,417 (4.9) | 57 (5.4) | 12 (3.4) | 0.13 |
| Non-CABG surgery, n (%) | 87/1,417 (6.1) | 68 (6.4) | 19 (5.4) | 0.48 |
| IABP, n (%) | 183/1,417(12.9) | 136 (12.8) | 47 (13.2) | 0.83 |
| IABP first (use of IABP prior to Impella), n (%) | 90/183 (49.2) | 55 (40.4) | 35 (74.5) | <0.001 |
| VA-ECMO use, n (%) | 594/1,417 (41.9) | 239 (22.5) | 355 (100) | <0.001 |
| VA-ECMO first (use of VA-ECMO prior to Impella), n (%) | 355/594 (59.8) | 0 (0) | 355 (100) | <0.001 |
| Ventricular assist device, n (%) | 4/1,417 (0.3) | 3 (0.3) | 1 (0.3) | 1 |
| Devise type of first Impella |  |  |  | 0.15 |
| Impella 2.5, n (%) | 27/1417 (1.9) | 21 (2.0) | 6 (1.7) |  |
| Impella CP, n (%) | 1379/1417 (97.3) | 1036 (97.6) | 343 (96.6) |  |
| Impella 5.0, n (%) | 6/1417 (0.4) | 3 (0.3) | 3 (0.8) |  |
| Impella 5.5, n (%) | 5/1417 (0.4) | 2 (0.2) | 3 (0.8) |  |
| Door to first Impella support, hour, median (IQR) | 1.8 (1.2-3.2) | 1.6 (1.1-2.7) | 2.7 (1.6-5.0) | <0.001 |
| Shock onset to first Impella support, hour, median (IQR) | 4.0 (2.1-6.8) | 3.7 (1.8-6.2) | 5.3 (3.1-8.6) | <0.001 |
| Shock onset to first Impella support ≤24 hour, n (%) | 1417 (100) | 1062 (100) | 355 (100) | - |
| Impella assist time, hour, median (IQR) | 96.1 (45.5-165.3) | 92.8 (44.5-157.3) | 119.2 (51.8-193.2) | <0.001 |
| Pragmatic DanGer shock criteria | 1417 (100) | 1062 (100) | 355 (100) | - |

Data are presented as n (%) or median (interquartile range). P values were calculated using the Mann–Whitney U test for continuous variables and the chi-square test for categorical variables to compare differences between the Impella-first and VA-ECMO first groups.

Abbreviations: CABG, coronary artery bypass grafting; IABP, intra-aortic balloon pump; IHCA, in-hospital cardiac arrest; IQR, interquartile range; LVEF, left ventricular ejection fraction; MC, mechanical complication; NSTEMI-CS, non–ST-segment elevation myocardial infarction complicated by cardiogenic shock; OHCA, out-of-hospital cardiac arrest; PCI, percutaneous coronary intervention; STEMI-CS, ST-segment elevation myocardial infarction complicated by cardiogenic shock; TIA, transient ischaemic attack; VA-ECMO, veno-arterial extracorporeal membrane oxygenation

**Table S5. Clinical events within 30 days after Impella implantation in patients with Eligible STEMI-CS, categorized by whether Impella or VA-ECMO was initiated first**

|  | Eligible STEMI-CS all  N = 1417 | Impella-first  N = 1062 (74.9%) | VA-ECMO-first  N = 355 (25.1%) | P value |
| --- | --- | --- | --- | --- |
| Death from any cause |  |  |  |  |
| Cumulative incidence at 30 days (95% CI) | 37.6 (34.9-40.2) | 32.6 (29.6-35.6) | 52.1 (46.5-57.2) | <0.001 |
| Median follow up period, median (IQR) | 24.0 (11.0-30.0) | 25.0 (12.0-30.0) | 19.0 (6.0-30.0) | <0.001 |
| Cardiac death |  |  |  |  |
| Cumulative incidence at 30 days (95% CI) | 31.2 (28.5-33.7) | 26.8 (23.8-29.6) | 44.3 (38.6-49.5) | <0.001 |
| Median follow up period, median (IQR) | 24.0 (11.0-30.0) | 25.0 (12.0-30.0) | 19.0 (6.0-30.0) | <0.001 |
| Non-cardiac death |  |  |  |  |
| Cumulative incidence at 30 days (95% CI) | 9.3 (7.4-11.1) | 8.0 (6.0-9.9) | 13.7 (8.9-18.3) | 0.009 |
| Median follow up period, median (IQR) | 24.0 (11.0-30.0) | 25.0 (12.0-30.0) | 19.0 (6.0-30.0) | <0.001 |
| Major bleeding |  |  |  |  |
| Cumulative incidence at 30 days (95% CI) | 28.9 (26.3-31.3) | 24.4 (21.7-27.1) | 42.7 (36.7-48.1) | <0.001 |
| Median follow up period, median (IQR) | 15.0 (2.0-30.0) | 18.5 (3.0-30.0) | 6.0 (1.0-30.0) | <0.001 |
| Impella-related bleeding |  |  |  |  |
| Cumulative incidence at 30 days (95% CI) | 15.6 (13.6-17.5) | 14.1 (11.9-16.3) | 20.4 (15.6-25.0) | 0.023 |
| Median follow up period, median (IQR) | 19.0 (4.0-30.0) | 21.0 (6.0-30.0) | 12.0 (2.0-30.0) | <0.001 |
| Intracranial bleeding |  |  |  |  |
| Cumulative incidence at 30 days (95% CI) | 3.3 (2.2-4.4) | 2.0 (1.0-2.9) | 7.7 (4.2-11.0) | <0.001 |
| Median follow up period, median (IQR) | 24.0 (11.0-30.0) | 25.0 (12.2-30.0) | 18.0 (5.0-30.0) | <0.001 |
| Limb ischaemia |  |  |  |  |
| Cumulative incidence at 30 days (95% CI) | 6.2 (4.9-7.6) | 5.4 (4.0-6.8) | 9.0 (5.6-12.2) | 0.042 |
| Median follow up period, median (IQR) | 22.0 (9.0-30.0) | 23.5 (11.0-30.0) | 17.0 (4.0-30.0) | <0.001 |
| Worsening renal failure |  |  |  |  |
| Cumulative incidence at 30 days (95% CI) | 12.2 (10.3-14.0) | 10.0 (8.1-11.9) | 19.3 (14.5-23.8) | <0.001 |
| Median follow up period, median (IQR) | 20.0 (6.0-30.0) | 22.0 (9.0-30.0) | 13.0 (3.0-30.0) | <0.001 |
| Ischaemic stroke |  |  |  |  |
| Cumulative incidence at 30 days (95% CI) | 4.9 (3.6-6.1) | 3.8 (2.5-5.0) | 8.7 (5.2-12.1) | 0.001 |
| Median follow up period, median (IQR) | 22.0 (10.0-30.0) | 24.0 (11.0-30.0) | 17.0 (5.0-30.0) | <0.001 |
| Ventricular arrhythmia |  |  |  |  |
| Cumulative incidence at 30 days (95% CI) | 6.0 (4.7-7.3) | 6.1 (4.6-7.6) | 5.8 (3.0-8.4) | 0.766 |
| Median follow up period, median (IQR) | 23.0 (9.0-30.0) | 24.0 (11.0-30.0) | 19.0 (5.0-30.0) | 0.0052 |
| Sepsis |  |  |  |  |
| Cumulative incidence at 30 days (95% CI) | 6.3 (4.8-7.7) | 6.4 (4.7-8.2) | 5.6 (2.8-8.3) | 0.88 |
| Median follow up period, median (IQR) | 23.0 (10.0-30.0) | 24.0 (11.0-30.0) | 18.0 (5.0-30.0) | 0.0013 |

Data are presented as cumulative incidence at 30 days with 95% confidence intervals or as median follow-up duration with interquartile ranges. P values for comparisons of cumulative incidence between the two groups were calculated using the log-rank test, and those for follow-up duration were calculated using the Mann–Whitney U test.

Abbreviations: CI, confidence interval; IQR, interquartile range; MC, mechanical complication; NSTEMI-CS, non–ST-segment elevation myocardial infarction complicated by cardiogenic shock; OHCA, out-of-hospital cardiac arrest; STEMI-CS, ST-segment elevation myocardial infarction complicated by cardiogenic shock; VA-ECMO, veno-arterial extracorporeal membrane oxygenation

**Table S6. Baseline characteristics and procedure details in OHCA patients, categorized by pre-hospital ROSC status**

|  | OHCA all  N = 992 | without ROSC  N = 354 (35.7%) | with ROSC  N = 638 (64.3%) | P value |
| --- | --- | --- | --- | --- |
| Age, median (IQR) | 64.0 (55.0-73.0) | 62.0 (52.0-71.0) | 66.0 (56.0-73.3) | <0.001 |
| Age >80, n (%) | 56/992 (5.6) | 9 (2.5) | 47 (7.4) | 0.002 |
| Male, n (%) | 882/992 (88.9) | 322 (91.0) | 560 (87.8) | 0.13 |
| Body mass index, kg/m2, median (IQR) | 24.2 (21.6-26.7) | 24.5 (21.7-27.0) | 23.9 (21.6-26.5) | 0.077 |
| Hypertension, n (%) | 525/992 (52.9) | 178 (50.3) | 347 (54.4) | 0.21 |
| Dyslipidemia, n (%) | 367/992 (37.0) | 112 (31.6) | 255 (40.0) | 0.009 |
| Diabetes mellitus, n (%) | 338/992 (34.1) | 104 (29.4) | 234 (36.7) | 0.02 |
| Current smoking, n (%) | 343/992 (34.6) | 118 (33.3) | 225 (35.3) | 0.54 |
| Prior myocardial infarction, n (%) | 153/992 (15.4) | 44 (12.4) | 109 (17.1) | 0.052 |
| History of heart failure, n (%) | 99/992 (10.0) | 20 (5.6) | 79 (12.4) | <0.001 |
| Chronic kidney disease, n (%) | 205/992 (20.7) | 69 (19.5) | 136 (21.3) | 0.50 |
| Haemodialysis, n (%) | 31/992 (3.1) | 6 (1.7) | 25 (3.9) | 0.054 |
| Prior stroke/TIA, n (%) | 64/992 (6.5) | 25 (7.1) | 39 (6.1) | 0.56 |
| IHCA, n (%) | 490/992 (49.4) | 198 (55.9) | 292 (45.8) | 0.002 |
| Systolic blood pressure, mmHg, median (IQR) | 80.0 (50.0-102.0) | 63.5 (0.0-92.3) | 85.0 (66.0-107.3) | <0.001 |
| Systolic blood pressure <100, mmHg, n (%) | 715/992 (72.1) | 284 (80.2) | 431 (67.6) | <0.001 |
| Diastolic blood pressure, mmHg, median (IQR) | 53.0 (27.0-72.0) | 41.5 (0.0-66.5) | 58.5 (40.0-73.0) | <0.001 |
| Heart rate, bpm, median (IQR) | 85.0 (60.0-104.0) | 78.0 (0.0-100.0) | 88.0 (70.0-108.0) | <0.001 |
| Creatinine, mg/dL, median (IQR) | 1.2 (1.0-1.5) | 1.2 (1.1-1.4) | 1.2 (1.0-1.5) | 0.77 |
| Creatinine >1.5 mg/dL, n (%) | 233/967 (24.1) | 68 (20.1) | 165 (26.3) | 0.031 |
| Albumin, g/dL, median (IQR) | 3.4 (2.8-3.8) | 3.2 (2.7-3.7) | 3.4 (2.9-3.8) | <0.001 |
| Lactate, mmol/L, median (IQR) | 11.1 (6.9-15.0) | 13.2 (9.2-16.3) | 9.7 (5.8-14.1) | <0.001 |
| Lactate ≥2.5 mmol/L, n (%) | 941/992 (94.9) | 345 (97.5) | 596 (93.4) | 0.006 |
| LVEF, %, median (IQR) | 20.0 (12.8-30.0) | 14.0 (10.0-20.0) | 25.0 (15.0-30.0) | <0.001 |
| LVEF <45%, n (%) | 973/992 (98.1) | 354 (100) | 619 (97.0) | 0.001 |
| Catecholamine use at initiation of Impella support |  |  |  |  |
| Epinephrine, n (%) | 343/992 (34.6) | 169 (47.7) | 174 (27.3) | <0.001 |
| Dobutamine, n (%) | 199/992 (20.1) | 50 (14.1) | 149 (23.4) | <0.001 |
| Norepinephrine, n (%) | 429/992 (43.2) | 108 (30.5) | 321 (50.3) | <0.001 |
| Dopamine, n (%) | 95/992 (9.6) | 19 (5.4) | 76 (11.9) | <0.001 |
| Any catecholamine, n (%) | 817/992 (82.4) | 282 (79.7) | 535 (83.9) | 0.097 |
| Use of pulmonary artery catheter, n (%) | 646/992 (65.1) | 230 (65.0) | 416 (65.2) | 0.94 |
| Hypoxic encephalopathy, n (%) | 258/992 (26.0) | 112 (31.6) | 146 (22.9) | 0.003 |
| PCI, n (%) | 922/992 (92.9) | 342 (96.6) | 580 (90.9) | <0.001 |
| CABG, n (%) | 33/992 (3.3) | 2 (0.6) | 31 (4.9) | <0.001 |
| Non-CABG surgery, n (%) | 38/992 (3.8) | 8 (2.3) | 30 (4.7) | 0.055 |
| IABP, n (%) | 85/992 (8.6) | 16 (4.5) | 69 (10.8) | <0.001 |
| IABP first (use of IABP prior to Impella), n (%) | 62/85 (72.9) | 13 (81.3) | 49 (71.0) | 0.41 |
| VA-ECMO use, n (%) | 622/992 (62.7) | 271 (76.6) | 351 (55.0) | <0.001 |
| VA-ECMO first (use of VA-ECMO prior to Impella), n (%) | 557/622 (89.5) | 266 (98.2) | 291 (82.9) | <0.001 |
| Ventricular assist device, n (%) | 3/992 (0.3) | 0 (0) | 3 (0.5) | 0.20 |
| Devise type of first Impella |  |  |  | 0.43 |
| Impella 2.5, n (%) | 24/992 (2.4) | 6 (1.7) | 18 (2.8) |  |
| Impella CP, n (%) | 950/992 (95.8) | 344 (97.2) | 606 (95.0) |  |
| Impella 5.0, n (%) | 8/992 (0.8) | 2 (0.6) | 6 (0.9) |  |
| Impella 5.5, n (%) | 10/992 (1.0) | 2 (0.6) | 8 (1.3) |  |
| Door to first Impella support, hour, median (IQR) | 2.0 (1.2-3.2) | 1.8 (1.2-2.5) | 2.1 (1.3-3.5) | <0.001 |
| Shock onset to first Impella support, hour, median (IQR) | 6.3 (4.2-9.8) | 6.0 (3.8-8.2) | 6.7 (4.3-10.5) | 0.011 |
| Shock onset to first Impella support ≤24 hour, n (%) | 906/992(91.3) | 332 (93.8) | 574 (90.0) | 0.041 |
| Impella assist time, hour, median (IQR) | 91.7 (42.2-159.2) | 94.3 (35.7-160.6) | 91.0 (43.3-158.6) | 0.98 |
| Pragmatic DanGer shock criteria | 806/992 (81.3) | 312 (88.1) | 494 (77.4) | <0.001 |
| Cause of OHCA |  |  |  | <0.001 |
| STEMI-CS | 733/992 (73.9) | 237 (66.9) | 496 (77.7) |  |
| MC | 17/992 (1.7) | 3 (0.8) | 14 (2.2) |  |
| NSTEMI | 242/992 (24.4) | 114 (32.2) | 128 (20.1) |  |

Data are presented as n (%) or median (interquartile range). P values were calculated using the Mann–Whitney U test for continuous variables and the chi-square test for categorical variables to compare differences between the with ROSC and without ROSC groups.

Abbreviations: CABG, coronary artery bypass grafting; IABP, intra-aortic balloon pump; IHCA, in-hospital cardiac arrest; IQR, interquartile range; LVEF, left ventricular ejection fraction; MC, mechanical complication; NSTEMI-CS, non–ST-segment elevation myocardial infarction complicated by cardiogenic shock; OHCA, out-of-hospital cardiac arrest; PCI, percutaneous coronary intervention; ROSC, return of spontaneous circulation; STEMI-CS, ST-segment elevation myocardial infarction complicated by cardiogenic shock; TIA, transient ischaemic attack; VA-ECMO, veno-arterial extracorporeal membrane oxygenation

**Table S7. Clinical events within 30 days after Impella implantation in patients with OHCA, categorized by pre-hospital ROSC status**

|  | OHCA all  N = 992 | without ROSC  N = 354 (35.7%) | with ROSC  N = 638 (64.3%) | P value |
| --- | --- | --- | --- | --- |
| Death from any cause |  |  |  |  |
| Cumulative incidence at 30 days (95% CI) | 51.3 (48.0-54.4) | 63.4 (57.8-68.2) | 44.5 (40.3-48.4) | <0.001 |
| Median follow up period, median (IQR) | 18.0 (4.0-30.0) | 12.0 (2.0-30.0) | 21.0 (8.0-30.0) | <0.001 |
| Cardiac death |  |  |  |  |
| Cumulative incidence at 30 days (95% CI) | 43.8 (40.4-47.0) | 56.0 (50.2-61.2) | 37.0 (32.9-40.9) | <0.001 |
| Median follow up period, median (IQR) | 18.0 (4.0-30.0) | 12.0 (2.0-30.0) | 21.0 (8.0-30.0) | <0.001 |
| Non-cardiac death |  |  |  |  |
| Cumulative incidence at 30 days (95% CI) | 13.1 (10.3-15.8) | 16.2 (10.7-21.4) | 11.7 (8.5-14.9) | 0.081 |
| Median follow up period, median (IQR) | 18.0 (4.0-30.0) | 12.0 (2.0-30.0) | 21.0 (8.0-30.0) | <0.001 |
| Major bleeding |  |  |  |  |
| Cumulative incidence at 30 days (95% CI) | 27.5 (24.4-30.5) | 31.9 (26.2-37.3) | 25.2 (21.6-28.7) | 0.029 |
| Median follow up period, median (IQR) | 10.0 (1.0-30.0) | 4.0 (1.0-23.8) | 14.0 (1.0-30.0) | <0.001 |
| Impella-related bleeding |  |  |  |  |
| Cumulative incidence at 30 days (95% CI) | 13.8 (11.4-16.1) | 17.4 (12.8-21.8) | 12.0 (9.3-14.6) | 0.039 |
| Median follow up period, median (IQR) | 15.0 (2.0-30.0) | 7.5 (1.0-29.0) | 17.0 (3.0-30.0) | <0.001 |
| Intracranial bleeding |  |  |  |  |
| Cumulative incidence at 30 days (95% CI) | 4.6 (3.0-6.2) | 5.4 (2.1-8.5) | 4.4 (2.6-6.2) | 0.83 |
| Median follow up period, median (IQR) | 19.0 (4.0-30.0) | 12.5 (2.0-30.0) | 21.5 (6.2-30.0) | <0.001 |
| Limb ischaemia |  |  |  |  |
| Cumulative incidence at 30 days (95% CI) | 4.5 (3.0-6.0) | 4.8 (2.0-7.4) | 4.5 (2.7-6.3) | 0.95 |
| Median follow up period, median (IQR) | 18.0 (4.0-30.0) | 12.0 (2.0-30.0) | 21.0 (6.0-30.0) | <0.001 |
| Worsening renal failure |  |  |  |  |
| Cumulative incidence at 30 days (95% CI) | 7.4 (5.5-9.2) | 8.5 (5.0-11.8) | 6.9 (4.6-9.0) | 0.39 |
| Median follow up period, median (IQR) | 17.0 (3.0-30.0) | 10.5 (2.0-30.0) | 20.0 (5.0-30.0) | <0.001 |
| Ischaemic stroke |  |  |  |  |
| Cumulative incidence at 30 days (95% CI) | 5.9 (4.1-7.7) | 7.1 (3.6-10.5) | 5.2 (3.2-7.3) | 0.25 |
| Median follow up period, median (IQR) | 17.0 (4.0-30.0) | 12.0 (2.0-30.0) | 20.0 (6.0-30.0) | <0.001 |
| Ventricular arrhythmia |  |  |  |  |
| Cumulative incidence at 30 days (95% CI) | 6.3 (4.5-8.0) | 6.8 (3.8-9.8) | 6.0 (3.9-8.0) | 0.53 |
| Median follow up period, median (IQR) | 17.5 (3.0-30.0) | 12.0 (2.0-30.0) | 20.5 (6.0-30.0) | <0.001 |
| Sepsis |  |  |  |  |
| Cumulative incidence at 30 days (95% CI) | 5.1 (3.4-6.7) | 4.9 (2.1-7.7) | 5.0 (3.1-7.0) | 0.77 |
| Median follow up period, median (IQR) | 17.0 (4.0-30.0) | 12.0 (2.0-30.0) | 20.0 (6.0-30.0) | <0.001 |

Data are presented as cumulative incidence at 30 days with 95% confidence intervals or as median follow-up duration with interquartile ranges. P values for comparisons of cumulative incidence between the two groups were calculated using the log-rank test, and those for follow-up duration were calculated using the Mann–Whitney U test.

Abbreviations: CI, confidence interval; IQR, interquartile range; MC, mechanical complication; NSTEMI-CS, non–ST-segment elevation myocardial infarction complicated by cardiogenic shock; OHCA, out-of-hospital cardiac arrest; ROSC, return of spontaneous circulation; STEMI-CS, ST-segment elevation myocardial infarction complicated by cardiogenic shock; VA-ECMO, veno-arterial extracorporeal membrane oxygenation

**Table S8. Baseline characteristics, and procedure details in patients with mechanical complications, particularly focusing on age stratification**

|  | MC all  N = 213 | Age ≤80  N = 159 (74.6%) | Age >80  N = 54 (25.4%) | P value |
| --- | --- | --- | --- | --- |
| Age, median (IQR) | 75.0 (69.0-81.0) | 72.0 (65.0-76.0) | 83.5 (82.0-86.0) | <0.001 |
| Male, n (%) | 127/213 (59.6) | 104 (65.4) | 23 (42.6) | 0.003 |
| Body mass index, kg/m2, median (IQR) | 23.1 (21.1-26.0) | 23.1 (20.8-26.0) | 23.1 (21.2-26.6) | 0.77 |
| Hypertension, n (%) | 138/213 (64.8) | 99 (62.3) | 39 (72.2) | 0.19 |
| Dyslipidemia, n (%) | 89/213 (41.8) | 67 (42.1) | 22 (40.7) | 0.86 |
| Diabetes mellitus, n (%) | 83/213 (39.0) | 65 (40.9) | 18 (33.3) | 0.33 |
| Current smoking, n (%) | 56/213 (26.3) | 53 (33.3) | 3 (5.6) | <0.001 |
| Prior myocardial infarction, n (%) | 65/213 (30.5) | 47 (29.6) | 18 (33.3) | 0.60 |
| History of heart failure, n (%) | 45/213 (21.1) | 29 (18.2) | 16 (29.6) | 0.076 |
| Chronic kidney disease, n (%) | 73/213 (34.3) | 55 (34.6) | 18 (33.3) | 0.87 |
| Haemodialysis, n (%) | 3/213 (1.4) | 2 (1.3) | 1 (1.9) | 0.75 |
| Prior stroke/TIA, n (%) | 17/213 (8.0) | 13 (8.2) | 4 (7.4) | 0.86 |
| IHCA, n (%) | 33/213 (15.5) | 24 (15.1) | 9 (16.7) | 0.78 |
| Systolic blood pressure, mmHg, median (IQR) | 90.0 (72.0-104.0) | 90.0 (72.0-106.0) | 88.0 (69.0-100.8) | 0.34 |
| Systolic blood pressure <100, mmHg, n (%) | 142/213 (66.7) | 104 (65.4) | 38 (70.4) | 0.50 |
| Diastolic blood pressure, mmHg, median (IQR) | 58.0 (45.0-69.0) | 58.0 (47.0-69.0) | 58.0 (40.0-69.5) | 0.51 |
| Heart rate, bpm, median (IQR) | 102.0 (83.5-116.0) | 101.0 (81.0-115.0) | 103.5 (89.8-119.3) | 0.24 |
| Creatinine, mg/dL, median (IQR) | 1.4 (1.0-2.2) | 1.3 (1.0-2.0) | 1.6 (1.1-2.6) | 0.11 |
| Creatinine >1.5 mg/dL, n (%) | 94/210 (44.8) | 65 (41.4) | 29 (54.7) | 0.092 |
| Albumin, g/dL, median (IQR) | 3.0 (2.7-3.4) | 3.0 (2.7-3.4) | 3.1 (2.6-3.6) | 0.68 |
| Lactate, mmol/L, median (IQR) | 3.7 (2.0-6.6) | 3.8 (2.0-6.6) | 3.7 (2.1-6.7) | 0.81 |
| Lactate ≥2.5 mmol/L, n (%) | 159/213 (74.6) | 117 (73.6) | 42 (77.8) | 0.54 |
| LVEF, %, median (IQR) | 45.0 (34.5-57.0) | 44.5 (33.8-55.0) | 45.0 (35.0-60.0) | 0.35 |
| LVEF <45%, n (%) | 148/213 (69.5) | 112 (70.4) | 36 (66.7) | 0.60 |
| Catecholamine use at initiation of Impella support |  |  |  |  |
| Epinephrine, n (%) | 27/213 (12.7) | 19 (11.9) | 8 (14.8) | 0.59 |
| Dobutamine, n (%) | 92/213 (43.2) | 74 (46.5) | 18 (33.3) | 0.09 |
| Norepinephrine, n (%) | 116/213 (54.5) | 88 (55.3) | 28 (51.9) | 0.66 |
| Dopamine, n (%) | 49/213 (23.0) | 36 (22.6) | 13 (24.1) | 0.83 |
| Any catecholamine, n (%) | 174/213 (81.7) | 132 (83.0) | 42 (77.8) | 0.39 |
| Use of pulmonary artery catheter, n (%) | 140/213 (65.7) | 109 (68.6) | 31 (57.4) | 0.14 |
| Hypoxic encephalopathy, n (%) | 7/213 (3.3) | 6 (3.8) | 1 (1.9) | 0.49 |
| PCI, n (%) | 96/213 (45.1) | 72 (45.3) | 24 (44.4) | 0.92 |
| CABG, n (%) | 52/213 (24.4) | 41 (25.8) | 11 (20.4) | 0.42 |
| Non-CABG surgery, n (%) | 158/213 (74.2) | 128 (80.5) | 30 (55.6) | <0.001 |
| IABP, n (%) | 57/213 (26.8) | 47 (29.6) | 10 (18.5) | 0.11 |
| IABP first (use of IABP prior to Impella), n (%) | 28/57 (49.1) | 22 (46.8) | 6 (60.0) | 0.45 |
| VA-ECMO use, n (%) | 108/213 (50.7) | 79 (49.7) | 29 (53.7) | 0.61 |
| VA-ECMO first (use of VA-ECMO prior to Impella), n (%) | 50/108 (46.3) | 38 (48.1) | 12 (41.4) | 0.54 |
| Ventricular assist device, n (%) | 1/213 (0.5) | 0 (0) | 1 (1.9) | 0.085 |
| Devise type of first Impella |  |  |  | 0.42 |
| Impella 2.5, n (%) | 6/213 (2.8) | 6 (3.8) | 0 |  |
| Impella CP, n (%) | 185/213 (86.9) | 137 (86.2) | 48 (88.9) |  |
| Impella 5.0, n (%) | 8/213 (3.8) | 5 (3.1) | 3 (5.6) |  |
| Impella 5.5, n (%) | 14/213 (6.6) | 11 (6.9) | 3 (5.6) |  |
| Door to first Impella support, hour, median (IQR) | 4.7 (2.1-23.5) | 4.8 (2.2-27.6) | 3.7 (1.9-15.9) | 0.16 |
| Shock onset to first Impella support, hour, median (IQR) | 10.3 (5.0-31.1) | 10.5 (5.1-30.5) | 10.1 (3.4-41.4) | 0.70 |
| Shock onset to first Impella support ≤24 hour, n (%) | 160/213(75.1) | 119 (74.8) | 41 (75.9) | 0.87 |
| Impella assist time, hour, median (IQR) | 111.6 (29.4-192.1) | 99.7 (26.0-190.5) | 115.1 (39.0-210.1) | 0.89 |
| Pragmatic DanGer shock criteria | 88/213 (41.3) | 64 (40.3) | 24 (44.4) | 0.59 |

Data are presented as n (%) or median (interquartile range). P values were calculated using the Mann–Whitney U test for continuous variables and the chi-square test for categorical variables to compare differences between the Age ≤80 and Age >80 groups.

Abbreviations: CABG, coronary artery bypass grafting; IABP, intra-aortic balloon pump; IHCA, in-hospital cardiac arrest; IQR, interquartile range; LVEF, left ventricular ejection fraction; MC, mechanical complication; NSTEMI-CS, non–ST-segment elevation myocardial infarction complicated by cardiogenic shock; OHCA, out-of-hospital cardiac arrest; PCI, percutaneous coronary intervention; STEMI-CS, ST-segment elevation myocardial infarction complicated by cardiogenic shock; TIA, transient ischaemic attack; VA-ECMO, veno-arterial extracorporeal membrane oxygenation

**Table S9. Clinical events within 30 days after Impella implantation in patients with mechanical complications**, **particularly focusing on age stratification**

|  | MC all  N = 213 | Age ≤80  N = 159 (74.6%) | Age >80  N = 54 (25.4%) | P value |
| --- | --- | --- | --- | --- |
| Death from any cause |  |  |  |  |
| Cumulative incidence at 30 days (95% CI) | 39.8 (32.7-46.2) | 35.5 (27.4-42.8) | 52.2 (36.3-64.1) | 0.041 |
| Median follow up period, median (IQR) | 30.0 (12.0-30.0) | 30.0 (13.5-30.0) | 24.0 (10.0-30.0) | 0.13 |
| Cardiac death |  |  |  |  |
| Cumulative incidence at 30 days (95% CI) | 34.5 (27.4-40.8) | 28.5 (20.7-35.4) | 51.2 (35.2-63.3) | 0.005 |
| Median follow up period, median (IQR) | 30.0 (12.0-30.0) | 30.0 (13.5-30.0) | 24.0 (10.0-30.0) | 0.13 |
| Non-cardiac death |  |  |  |  |
| Cumulative incidence at 30 days (95% CI) | 8.0 (3.7-12.2) | 9.8 (4.3-15.0) | 1.9 (0.0-5.5) | 0.18 |
| Median follow up period, median (IQR) | 30.0 (12.0-30.0) | 30.0 (13.5-30.0) | 24.0 (10.0-30.0) | 0.13 |
| Major bleeding |  |  |  |  |
| Cumulative incidence at 30 days (95% CI) | 17.7 (12.1-22.9) | 18.6 (12.1-24.7) | 15.1 (4.0-24.8) | 0.50 |
| Median follow up period, median (IQR) | 22.0 (8.0-30.0) | 24.0 (8.0-30.0) | 21.0 (5.2-30.0) | 0.32 |
| Impella-related bleeding |  |  |  |  |
| Cumulative incidence at 30 days (95% CI) | 6.0 (2.6-9.2) | 6.6 (2.5-10.5) | 4.2 (0.0-9.7) | 0.51 |
| Median follow up period, median (IQR) | 28.0 (10.0-30.0) | 30.0 (10.5-30.0) | 23.0 (8.0-30.0) | 0.18 |
| Intracranial bleeding |  |  |  |  |
| Cumulative incidence at 30 days (95% CI) | 2.5 (0.3-4.7) | 2.7 (0.1-5.3) | 1.9 (0.0-5.5) | 0.83 |
| Median follow up period, median (IQR) | 30.0 (12.0-30.0) | 30.0 (12.5-30.0) | 24.0 (10.0-30.0) | 0.13 |
| Limb ischaemia |  |  |  |  |
| Cumulative incidence at 30 days (95% CI) | 4.7 (1.6-7.6) | 3.5 (0.4-6.4) | 8.7 (0.1-16.5) | 0.14 |
| Median follow up period, median (IQR) | 29.0 (10.0-30.0) | 30.0 (12.5-30.0) | 22.5 (5.2-30.0) | 0.023 |
| Worsening renal failure |  |  |  |  |
| Cumulative incidence at 30 days (95% CI) | 12.0 (7.2-16.5) | 11.0 (5.7-16.0) | 14.9 (3.9-24.7) | 0.46 |
| Median follow up period, median (IQR) | 27.0 (9.0-30.0) | 29.0 (9.5-30.0) | 22.5 (5.2-30.0) | 0.10 |
| Ischaemic stroke |  |  |  |  |
| Cumulative incidence at 30 days (95% CI) | 7 (3.1-10.8) | 6.6 (2.3-10.8) | 8.5 (0.0-17.5) | 0.94 |
| Median follow up period, median (IQR) | 28.0 (11.0-30.0) | 30.0 (11.0-30.0) | 22.5 (10.0-30.0) | 0.080 |
| Ventricular arrhythmia |  |  |  |  |
| Cumulative incidence at 30 days (95% CI) | 1.7 (0.0-3.7) | 0.7 (0.0-2.1) | 5.0 (0.0-11.6) | 0.078 |
| Median follow up period, median (IQR) | 30.0 (12.0-30.0) | 30.0 (13.5-30.0) | 22.5 (8.5-30.0) | 0.05 |
| Sepsis |  |  |  |  |
| Cumulative incidence at 30 days (95% CI) | 6 (2.0-9.9) | 6.0 (1.5-10.2) | 6.4 (0.0-14.9) | 0.95 |
| Median follow up period, median (IQR) | 29.0 (11.0-30.0) | 30.0 (11.5-30.0) | 23.5 (8.5-30.0) | 0.11 |

Data are presented as cumulative incidence at 30 days with 95% confidence intervals or as median follow-up duration with interquartile ranges. P values for comparisons of cumulative incidence between the two groups were calculated using the log-rank test, and those for follow-up duration were calculated using the Mann–Whitney U test.

Abbreviations: CI, confidence interval; IQR, interquartile range; MC, mechanical complication; NSTEMI-CS, non–ST-segment elevation myocardial infarction complicated by cardiogenic shock; OHCA, out-of-hospital cardiac arrest; STEMI-CS, ST-segment elevation myocardial infarction complicated by cardiogenic shock; VA-ECMO, veno-arterial extracorporeal membrane oxygenation
